# Identification and characterization of differentially expressed genes in *Caenorhabditis elegans* in response to pathogenic and nonpathogenic *Stenotrophomonas maltophilia*

**DOI:** 10.1186/s12866-020-01771-1

**Published:** 2020-06-19

**Authors:** Leah J. Radeke, Michael A. Herman

**Affiliations:** School of Biological Sciences, University of Nebraska-Lincoln, Lincoln, NE 68588 USA

**Keywords:** *Caenorhabditis elegans*, *Stenotrophomonas maltophilia*, Differential expression, Innate immune response, Bacteria

## Abstract

**Background:**

*Stenotrophomonas maltophilia* is an emerging nosocomial pathogen that causes infection in immunocompromised patients. *S. maltophilia* isolates are genetically diverse, contain diverse virulence factors, and are variably pathogenic within several host species. Members of the *Stenotrophomonas* genus are part of the native microbiome of *C. elegans*, being found in greater relative abundance within the worm than its environment, suggesting that these bacteria accumulate within *C. elegans*. Thus, study of the *C. elegans-Stenotrophomonas* interaction is of both medical and ecological significance. To identify host defense mechanisms, we analyzed the *C. elegans* transcriptomic response to *S. maltophilia* strains of varying pathogenicity: K279a, an avirulent clinical isolate, JCMS, a virulent strain isolated in association with soil nematodes near Manhattan, KS, and JV3, an even more virulent environmental isolate.

**Results:**

Overall, we found 145 genes that are commonly differentially expressed in response to pathogenic *S. maltophilia* strains, 89% of which are upregulated, with many even further upregulated in response to JV3 as compared to JCMS. There are many more JV3-specific differentially expressed genes (225, 11% upregulated) than JCMS-specific differentially expressed genes (14, 86% upregulated), suggesting JV3 has unique pathogenic mechanisms that could explain its increased virulence. We used connectivity within a gene network model to choose pathogen-specific and strain-specific differentially expressed candidate genes for functional analysis. Mutations in 13 of 22 candidate genes caused significant differences in *C. elegans* survival in response to at least one *S. maltophilia* strain, although not always the strain that induced differential expression, suggesting a dynamic response to varying levels of pathogenicity.

**Conclusions:**

Variation in observed pathogenicity and differences in host transcriptional responses to *S. maltophilia* strains reveal that strain-specific mechanisms play important roles in *S. maltophilia* pathogenesis. Furthermore, utilizing bacteria closely related to strains found in *C. elegans* natural environment provides a more realistic interaction for understanding host-pathogen response.

## Background

*Stenotrophomonas maltophilia* is a Gram-negative, nosocomial pathogen that can cause infection in immunocompromised patients. *S. maltophilia* is often found in patients with cystic fibrosis and lung cancer, and is associated with infections such as pneumonia, endocarditis, bacteremia, and meningitis [1]. Although not highly virulent, *S. maltophilia* is multi-drug resistant and capable of forming biofilms [2, 3] thus developing treatment methods for this pathogen is becoming an increasing concern. *S. maltophilia* is ubiquitous within the environment, commonly found in aqueous sources, soils, and associated with plant roots, and can also be isolated in hospitals from water sources and medical devices [1, 4].

Sequencing and functional analyses have identified both similarities and differences in virulence factors such as antibiotic resistance and quorum sensing mechanisms in clinical and environmental isolates of *S. maltophilia* [5–10]. Therefore, strain diversity appears to result in different virulence mechanisms and pathogenic potential [5, 11]. Although studies have identified virulence factors within *S. maltophilia* genomes, phenotypic analysis using host species is rarely performed, and mechanisms of host responses are poorly understood. Therefore, we recently established *Caenorhabditis elegans* as a model to study host responses to *S. maltophilia* infection [12].

*C. elegans* is an excellent genetic model organism for studying many biological processes, including development, neurobiology, and innate immunity. *C. elegans* are bacterivores and can be found in the natural environment in decaying fruits and stems where they are in contact with many bacterial species. Recent studies have found that *Stenotrophomonas* is one of the most abundant genera of bacteria found in the native microbiome of *C. elegans* [13–15]. Furthermore, *Stenotrophomonas* is found in higher abundance within the microbiome than in rotting substrates where *C. elegans* are found [14, 15], suggesting that it colonizes and accumulates in the intestine, a common signature of pathogenesis in *C. elegans* [16, 17]. In fact, many of these *Stenotrophomonas* isolates were found to be detrimental to the health of *C. elegans*, while few were found to be beneficial [14, 18]. This is consistent with previous observations that *S. maltophilia* strains show varying pathogenicity to *C. elegans,* amoeba (*Dictyostelium discoideum* and *Acanthamoeba castellanii*), and zebrafish [6, 8, 12]. This suggests that *S. maltophilia* strains utilize different virulence mechanisms that result in different host responses.

Many innate immune pathways in *C. elegans* are conserved from invertebrates to mammals, making it an excellent model for studying pathogen-host interactions and innate immunity. Briefly, the p38 mitogen-activated protein kinase (MAPK) pathway plays a role in defense against several pathogens, including *S. maltophilia*, *Pseudomonas aeruginosa*, *Staphylococcus aureus*, and *Salmonella enterica* [12, 19–21]. In addition, activation of the insulin-like signaling pathway decreases bacterial packing, suggesting that regulation of genes by the downstream transcription factor DAF-16 defends against accumulation of bacteria in the intestine [16]. Analyses of mutations affecting genes in these pathways have identified downstream proteins involved in pathogen defense, such as lysozymes, C-lectins, and CUB-domain containing proteins [22, 23].

Although many important innate immune pathways and effectors have been identified, there are differences in responses to different bacterial pathogens. For example, one study comparing responses to intestinal pathogens *Serratia marcescens*, *Enterococcus faecalis*, and *Photorhabdus luminescens* found that only 11% of genes in *C. elegans* where commonly differentially expressed in response to all three species [24]. This phenomenon could be due to species-specific responses to different pathogens, or the ability of bacteria to manipulate different host responses. Therefore, it is essential to study a variety of pathogens in order to fully understand the complexity of genetic mechanisms underlying pathogen defense. Finally, studying bacteria that have been identified as part of the microbiome of *C. elegans*, or their close relatives, more closely approximates natural interactions and therefore a more realistic response to the bacteria.

We previously used a microarray approach to identify gene expression patterns in response to *Escherichia coli* OP50 and *S. maltophilia* strains K279a and JCMS [25]. We found that the genetic response is more specific to the type of *C. elegans-*bacterial interaction rather than bacteria species or strain. Here, we further explore these responses using RNA sequencing to identify and characterize the genetic responses of *C. elegans* to several different *S. maltophilia* strains of varying levels of pathogenicity. Specifically, we performed transcriptomic analysis on *C. elegans* following exposure to either *E. coli* OP50 or one of three *S. maltophilia* strains: two pathogenic environmental isolates, JCMS and JV3, and one nonpathogenic clinical isolate, K279a. Using this experimental set-up, we discovered responses that are common to both pathogenic *S. maltophilia* strains and responses that are strain-specific based on the level of virulence of each strain. We also identified candidate genes involved in both the common and strain-specific responses and determined that several candidate genes were important for survival of *C. elegans* upon exposure to *S. maltophilia*.

## Results

**Fig. 1:**
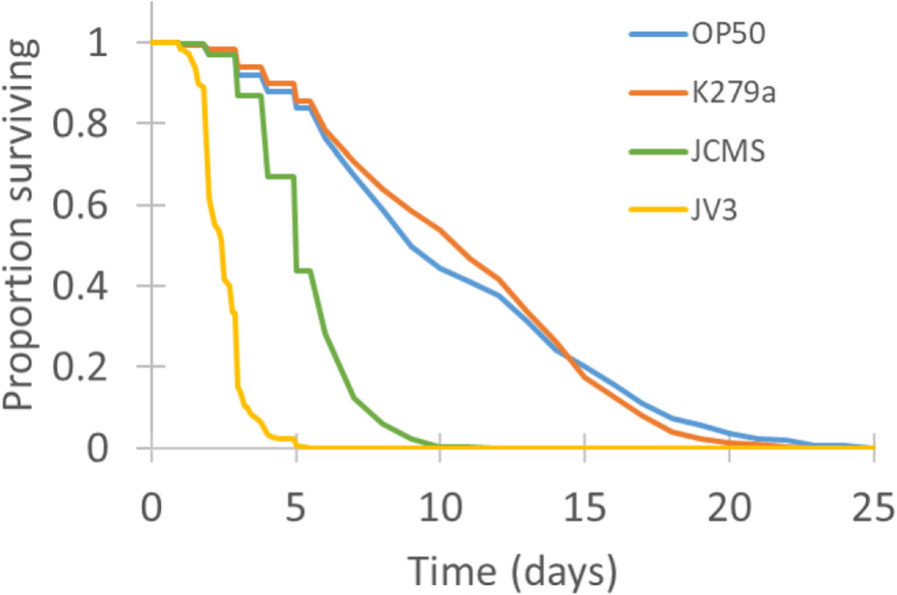
*S. maltophilia* strains show varying pathogenicity to *C. elegans.* Survivorship of *wild-type* nematodes on *S. maltophilia* JCMS, K279a, JV3, and *E. coli* OP50. Survival estimates were determined using Kaplan-Meier estimates generated in R. This data contains all *wild-type* data collected from experiments in this study, representing 23 individual experiments and *n* = 516–615 for each bacterial treatment. Sample sizes, hazard ratios and *p*-values generated form Cox proportional hazards tests are shown in Table 3

We used survival as an indicator of bacterial pathogenicity to *C. elegans.* Survival analyses showed that *S. maltophilia* strains K279a, JCMS, and JV3, display differing levels of pathogenicity to *C. elegans*. *S. maltophilia* K279a, a clinical isolate of *S. maltophilia*, is not pathogenic, as worms fed K279a have similar bacterial load and survival as do worms fed the standard lab food *E. coli* OP50 [12] (Fig. 1). However, *S. maltophilia* JCMS, a strain isolated in association with soil nematodes and *S. maltophilia* JV3, another environmental isolate closely related to JCMS, are both detrimental to the survival of *C. elegans* (Fig. 1). We used the Cox proportional hazards test to quantify these differences by calculating the hazard, or the probability of a nematode dying at a given time, for each bacterial treatment. Hazards ratios are used to compare relative hazards of different conditions, in this case bacteria, where ratios greater than one indicate treatments that are more detrimental, or hazardous, to the health of *C. elegans*; whereas hazard ratios less than one indicate more beneficial conditions. *C. elegans* exposed to JCMS have a hazard of 6.66 (±0.07), meaning they are 6.66 times more likely to die than *C. elegans* exposed to OP50, whereas *C. elegans* exposed to JV3 are 95.64 (±0.08) times more likely to die than *C. elegans* fed OP50. We performed a transcriptomic analysis to discover the genes underlying the response of *C. elegans* to strains of *S. maltophilia* of varying pathogenicity to provide a more comprehensive understanding of *C. elegans*-pathogen interactions.

### *C. elegans* exhibit common and strain-specific responses to *S. maltophilia*

To investigate transcriptomic responses to *S. maltophilia*, RNA-sequencing was performed after 12 h of exposure to pathogenic *S. maltophilia* JCMS or JV3, or nonpathogenic *S. maltophilia* K279a or *E. coli* OP50. The 12-h time point was chosen based on previous observations that accumulation of bacteria occurs by this time [12] but *S. maltophilia* JV3-induced mortality has not yet begun (Fig. 1). In addition, other groups have identified transcriptional changes at 4–8 h of exposure to pathogens, including *S. aureus*, *Bacillus thuringiensis,* and *P. aeruginosa* [23, 26, 27]. Therefore, at 12 h, pathogen recognition has begun, but transcriptional changes associated with aging and mortality, which correlate with a decreased immune response [28], should not complicate interpretation of data.

**Fig. 2:**
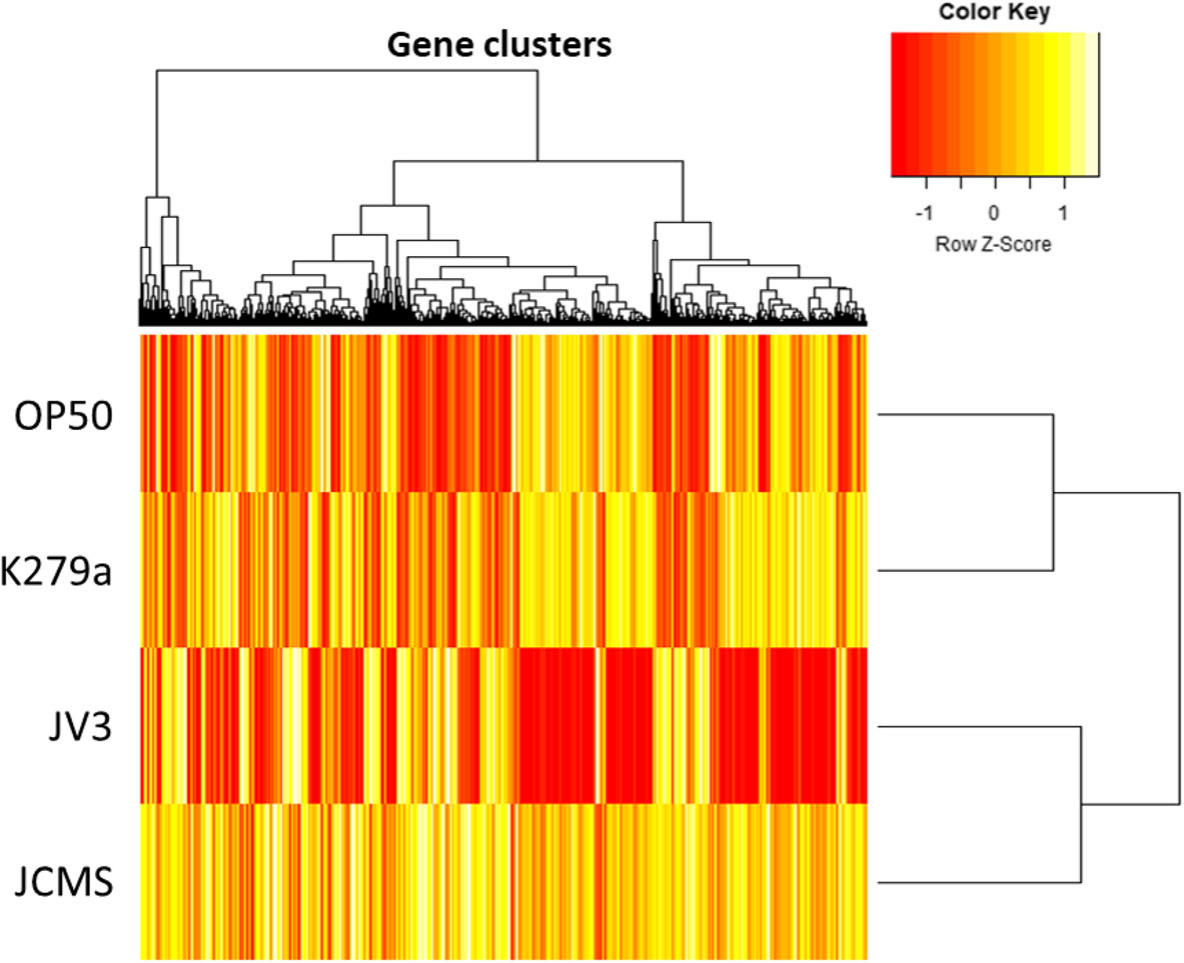
Heatmap of *C. elegans* differentially expressed genes in response to *S. maltophilia.* Differentially expressed genes from RNA-sequencing include genes with fold-change > 2 and FDR-adjusted *p*-value < 0.05 between any treatment comparisons. Heatmap was generated with log transformed FPKM values and visualized using the heatmap.2 function in gplots package in R. Dendrogram on the y-axis represents degree of similarity of treatments based on gene expression profiles. Dendrogram on the x-axis represents degree of similarity of gene clusters based on expression profile across treatments. Gene expression is color coded, with red indicating lower expression and yellow indicating higher expression. The scaling option was used so that each gene is individually normalized across treatments

**Fig. 3:**
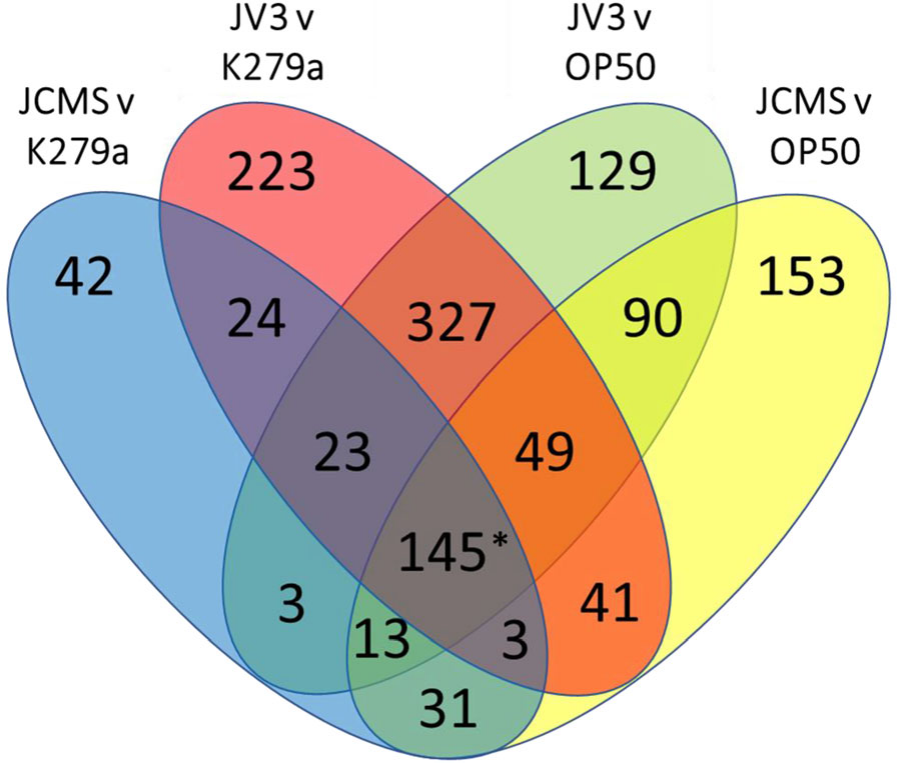
*C. elegans* expresses a common set of 145 genes in response to pathogenic *S. maltophilia* strains. Differential expression was determined between each pathogenic and nonpathogenic comparison, with the number of significantly differentially expressed genes indicated between each set of comparisons. Genes included are differentially expressed between the specified treatments with fold-change > 2 and FDR-adjusted *p*-value < 0.05. 145 genes were commonly differentially expressed between all pathogenic (JCMS and JV3) and nonpathogenic (K279a and OP50) treatments, or the common pathogenic *S. maltophilia* response (CPSR), indicated by the asterisk

Overall gene expression patterns were analyzed using a heatmap of genes that were significantly differentially expressed between any two treatments (Fig. 2; Supplemental Table 1, Additional File 1). Transcripts were considered differentially expressed if they had a false discovery rate (FDR)-adjusted *p*-value of less than 0.05 and an absolute fold change greater than two. Gene expression profiles showed clustering of nonpathogenic (K279a and *E. coli* OP50) and pathogenic (JCMS and JV3) treatments (Fig. 2). Although there are differences between expression profiles of the nonpathogenic strains, suggesting the existence of a species-specific response, the expression profiles of the nonpathogenic treatments were more similar than that of the pathogenic treatments (Fig. 2). Therefore, to identify the common response to pathogenic *S. maltophilia*, we compared differentially expressed genes in *C. elegans* between pathogenic and nonpathogenic treatments (Fig. 3). In total, 1296 genes were significantly differentially expressed when comparing worms fed any pathogenic (JV3 and JCMS) to any nonpathogenic (K279a and *E. coli* OP50) strain, with 11% (145) commonly differentially expressed between all pathogenic and nonpathogenic comparisons (Fig. 3, Supplemental Table 2; Supplemental Table 3, Additional File 1) These most likely represent a core set of genes that are regulated upon exposure to pathogenic *S. maltophilia* and are therefore referred to as the “common pathogenic *S. maltophilia* response” (CPSR). Because these genes are differentially expressed in response to pathogenic vs nonpathogenic strains of the same species, this should remove general responses to *S. maltophilia* and represent genes specifically involved in pathogen response to *S. maltophilia*. Of the 145 CPSR genes, 129 (89%) were upregulated in response to the pathogenic strains as compared to the nonpathogenic strains, whereas 15 (10%) were downregulated (Supplemental Table 3, Additional File 1). One gene, *lys-10*, is upregulated in response to the pathogenic strains compared to OP50 but downregulated in response to pathogenic strains compared to K279a. Interestingly, most upregulated genes, 90 of 129, were even further upregulated in response to JV3 as compared to JCMS. Because JV3 is more virulent than JCMS, this suggests that the level of virulence influences the expression of *S. maltophilia*-induced genes.

A gene ontology (GO) enrichment analysis was performed on all CPSR genes using the Database for Annotation, Visualization and Integrated Discovery (DAVID) [29, 30] to identify common cellular components, biological processes, and molecular functions of these genes. The terms “biological process of innate immune response” (FDR = 1.48E-51), “biological process of defense to Gram-negative bacterium” (FDR = 4.19E-11), “molecular function of carbohydrate binding” (FDR = 1.84E-4), and “cellular component of membrane raft” (FDR = 8.13E-20) were all significantly enriched among the CPSR genes (Table 1). We also analyzed GO enrichment for the up- and down-regulated genes separately. Enriched GO terms for the upregulated CPSR genes were very similar to those for all CPSR genes (Supplemental Table 6, Additional File 2), whereas analysis of downregulated genes resulted in no significant GO terms with FDR < 0.05, possibly due to the small number of downregulated CPSR genes (15).

**Table 1:** Innate immune response GO terms significantly enriched in common pathogenic *S. maltophilia* response (CPSR) genes

GO category	Term	Count	%	FDR
Biological process	response to stimulus	60	43.48	4.19E-11
	response to stress	55	39.86	3.47E-31
	defense response	52	37.68	2.33E-46
	innate immune response	50	36.23	1.48E-51
	defense response to bacterium	16	11.59	6.58E-12
	defense response to Gram-negative bacterium	14	10.14	4.19E-11
Molecular function	carbohydrate binding	12	8.70	1.84E-04
Cellular component	membrane raft	17	12.32	8.13E-20

Gene ontology (GO) enrichment analysis was performed on the CPSR genes using DAVID Bioinformatics Resources 6.8. Of the 145 CPSR genes, 138 were identified in DAVID and considered for analysis. GO analysis identifies terms relating to the biological process, molecular function, or cellular component that are significantly enriched among a list of genes. Indented terms indicate child terms, or subcategories, of the term listed above, with the parent term left-aligned. Note that the degree of indention of each term does not reflect absolute GO term level within each category. Count is the number of genes corresponding to each GO term. Percent is the count/138 total analyzed. FDR is the false discovery rate-corrected EASE enrichment score to account for multiple testing. Only terms with FDR <0.05 and the most descriptive term for each unique gene list are shown

To identify JV3- and JCMS-specific responses, we identified genes that were differentially expressed in response to JV3 and JCMS as compared to all other strains. We found 31 genes differentially expressed in response to JCMS vs the nonpathogenic strains and 327 genes differentially expressed in response to JV3 vs the nonpathogenic strains (Fig. 3). We found that 14 of the 31 JCMS vs nonpathogenic strains genes were also differentially expressed between JV3 and JCMS (Supplemental Table 4, Additional File 1). These genes are specifically regulated upon exposure to *S. maltophilia* JCMS and are therefore referred to as the “JCMS-specific response” (JSR). Of the 14 JSR genes, 12 are upregulated in response to JCMS as compared to all other strains, whereas two are downregulated (Supplemental Table 4, Additional File 1).

We found that 225 of the 327 JV3 vs nonpathogenic strain genes were also differentially expressed between JV3 and JCMS (Fig. 3; Supplemental Table 5, Additional File 1). These genes are specifically regulated upon exposure to *S. maltophilia* JV3 and are referred to as the “JV3-specific response” (VSR). Although most CPSR genes are upregulated in response to JV3, a majority (89%) of the VSR genes are downregulated in response to JV3 as compared to the other strains (Supplemental Table 5, Additional File 1). This suggests that one virulence mechanism employed by JV3 may be to reduce expression of a variety of host genes. GO enrichment analyses of these genes reveals enrichment of several metabolic processes and enzymes, including “biological process of flavonoid glucuronidation” (FDR = 3.02E-09), “biological process of oxidation-reduction process” (FDR = 0.0433), “molecular function of glucuronosyltransferase activity” (FDR = 9.7E-06), and “molecular function of carboxylic ester hydrolase activity” (FDR = 7.24E-04) (Table 2).

**Table 2:** Metabolism and enzyme GO terms significantly enriched in *S. maltophilia* JV3-specific response (VSR) genes

GO category	Term	Count	%	FDR
Biological process	single-organism metabolic process	49	22.68	2.47E-06
	Small molecule metabolic process	27	12.5	5.27E-04
	organic acid metabolic process	22	10.19	1.64E-05
	carboxylic acid metabolic process	21	9.72	2.21E-05
	monocarboxylic acid metabolic process	18	8.33	8.53E-07
	flavonoid glucuronidation	14	6.48	3.02E-09
	flavonoid biosynthetic process	14	6.48	3.02E-09
	oxidation-reduction process	21	9.72	0.0433
	transition metal ion transport	7	3.24	0.0468
Molecular function	catalytic activity	89	41.20	0.00174
	transferase activity, transferring glycosyl groups	17	7.87	0.00203
	transferase activity, transferring hexosyl groups	14	6.48	1.76E-07
	glucuronosyltransferase activity	12	5.56	9.70E-06
	UDP-glycosyltransferase activity	16	7.41	0.00155
	carboxylic ester hydrolase activity	11	5.09	7.24E-04
Cellular component	extracellular region	20	9.26	0.0295

Gene ontology (GO) enrichment analysis was performed on the VSR genes using DAVID Bioinformatics Resources 6.8. Of the 225 VSR genes, 216 were identified in DAVID and considered for analysis. GO analysis identifies terms relating to the biological process, molecular function, or cellular component that are significantly enriched among a list of genes. Indented terms indicate child terms, or subcategories, of the term listed above, with the parent term left-aligned. Note that the degree of indention of each term does not reflect absolute GO term level within each category. Count is the number of genes corresponding to each GO term. Percent is the count/216 total analyzed. FDR is the false discovery rate-corrected EASE enrichment score to account for multiple testing. Only terms with FDR <0.05 and the most descriptive term for each unique gene list are shown

Again, we also analyzed GO enrichment for the up- and down-regulated VSR genes separately. Downregulated enriched GO terms were very similar to those for all VSR genes (Supplemental Table 7, Additional File 2), whereas analysis of upregulated genes resulted in no significant GO terms with and FDR < 0.05, possibly due to the small number of upregulated VSR genes.

### Gene network analysis to prioritize important response genes

We next wanted to determine whether the CPSR, JSR, and VSR genes are important for the response to both pathogenic *S. maltophilia* strains (CPSR genes) or to specific strains of *S. maltophilia* (JSR and VSR genes). To do this, we utilized WormNet, a probabilistic gene network model, to prioritize genes for functional analysis [31]. WormNet uses both direct physical and/or genetic interactions as well as inferred interactions to create a gene network that comprises 75.4% (15,139 genes) of the *C. elegans* genome, resulting in 999,367 functional linkages [31]. Previously, gene networks have been used to identify genes essential for *C. elegans* development and survival under standard conditions, as well as identification of genes associated with particular diseases [32, 33]. In addition, we previously found this method to be helpful to identify functionally important *S. maltophilia*-induced genes [25]. Therefore, we hypothesize that the most connected genes within the gene network play a significant role in *S. maltophilia* response and are therefore better candidates for functional analyses. In addition to gene network connectivity, we preferentially chose genes for functional analysis with available alleles, either from the *Caenorhabditis* Genetics Center (CGC) or previously generated in our lab, and generated mutant alleles using CRISPR/Cas9 for additional genes that were expressed at higher levels.

**Fig. 4:**
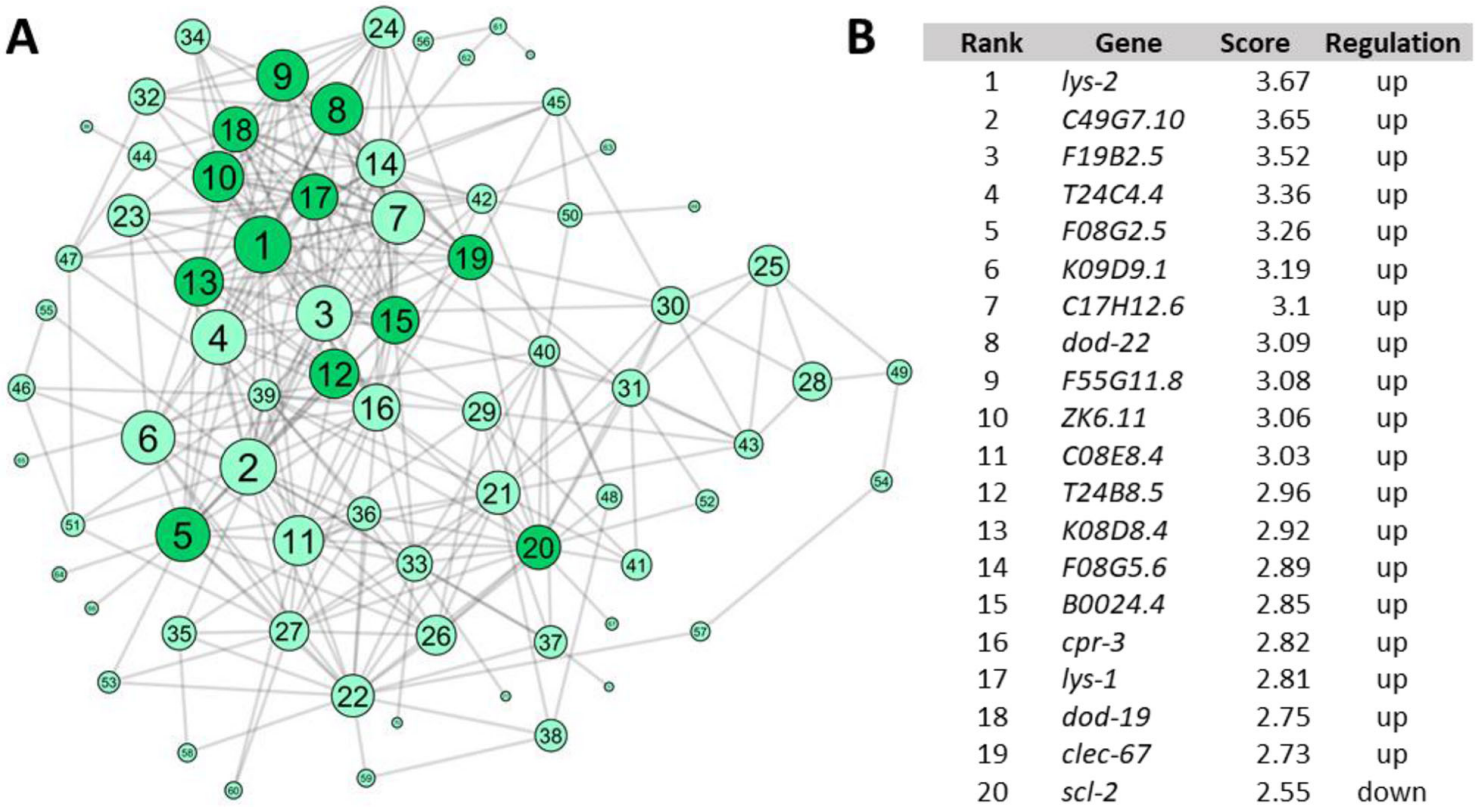
Gene network analysis was used to prioritize CPSR genes. WormNet v2, a probabilistic functional gene network model, was queried with the 145 genes that were differentially expressed in response to non-pathogenic vs. pathogenic strains. 73 of the 145 genes are connected to one another (AUC = 0.6942, *p* = 1.8137e-16). **a** Network visualized using Cytoscape 3.5.1. Green circles represent individual genes and grey lines represent known or predicted connections between genes. Genes are numbered and sized based on rank (Supplemental Table 8, Additional File 3); darker green circles indicate genes that were chosen for functional analysis. **b** Top 20 ranked genes. Rank is determined based on score, which is calculated based on number of connections and the strength of evidence for those connections. Regulation indicates direction of differential expression in response to pathogenic compared to nonpathogenic strains

Of the 145 CPSR genes, 73 were connected within the gene network with an AUC of 0.6972 (*p* = 1.8E-16) (Fig. 4; Supplemental Table 8, Additional File 3). The AUC is the area under the receiver operating characteristic (ROC) curve and provides a measure for the recovery of true-positive genes as compared to false-positive genes [31]. A random network would have an AUC of 0.5, whereas a network representing perfect prediction of all connections would have an AUC of one; therefore, an AUC of 0.6972 (*p* = 1.81E-16) suggests relatively high predictive power of gene connections. Each connected gene is ranked based on the number of connections as well as the strength of the evidence for those connections [32], with some of the highest-ranking CPSR genes including *lys-1*, *lys-2*, *dod-22*, *dod-19*, and *clec-67* (Supplemental Table 8, Additional File 3). Previous studies have identified these genes as downstream effectors of defense pathways or directly involved in response to bacterial pathogen challenge [12, 22, 34, 35]. Mutant alleles were available for these genes, along with alleles of several other genes highly connected within the network, including *F55G11.8, ZK6.11, T24B8.5*, and *scl-2*. In addition, we had previously generated mutant alleles affecting *K08D8.4, B0024.4*, and *F08G2.5* using CRISPR/Cas9 (Additional File 4).

**Fig. 5:**
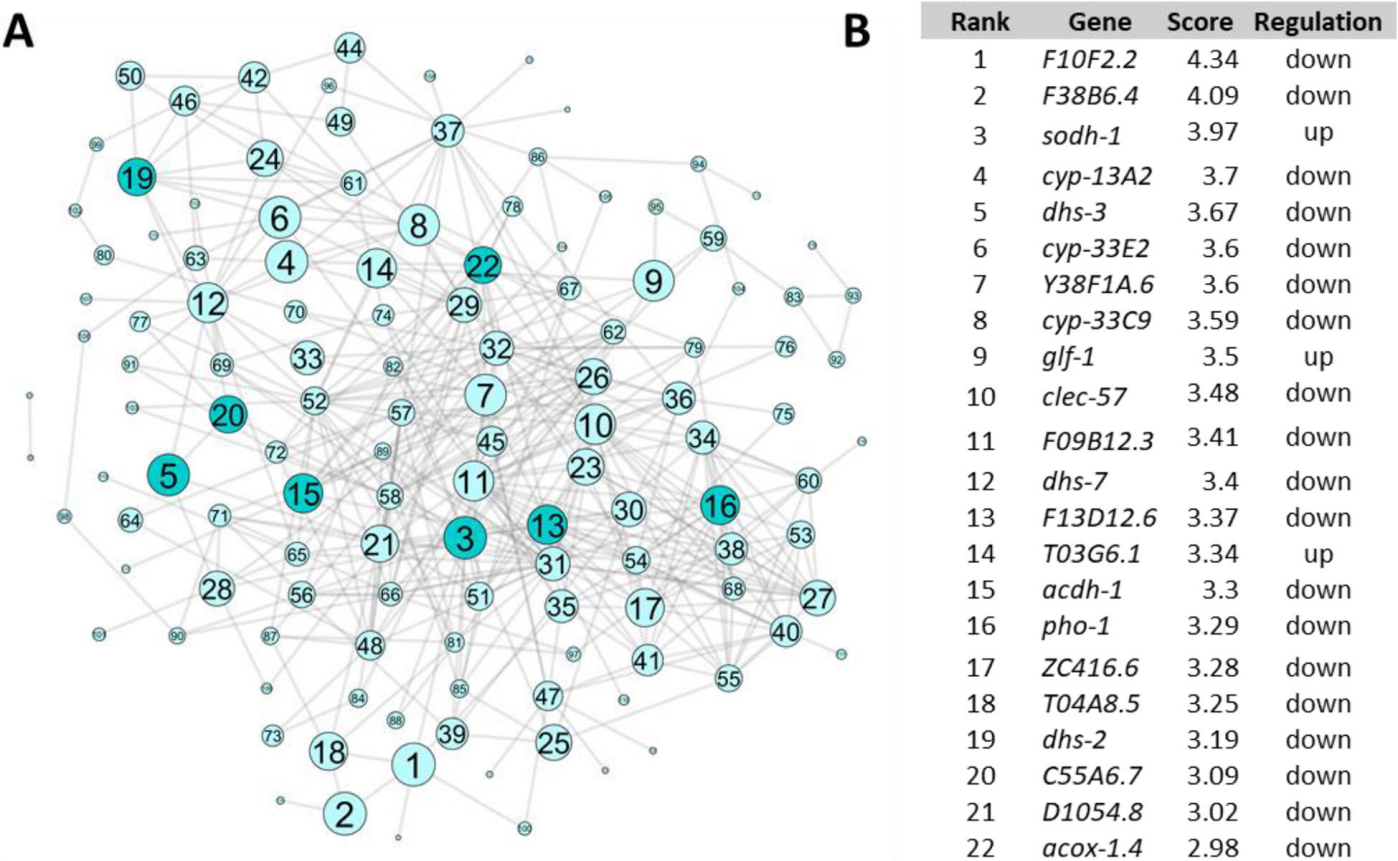
Gene network analysis was used to prioritize VSR genes. WormNet v2, a probabilistic functional gene network model was queried with the 225 JV3-specific response (VSR) genes. 128 of the 225 genes are connected to one another (AUC = 0.6776, *p* = 2.81E-22). **a** Network was visualized using Cytoscape 3.5.1. Blue circles represent individual genes and grey lines represent known or predicted connections between genes. Genes are numbered and sized based on rank (Supplemental Table 9, Additional File 3); darker blue circles indicate genes that were chosen for functional analysis. **b** Top 22 ranked genes. Rank is determined based on score, which is calculated based on number of connections and the strength of evidence for those connections. Regulation indicates direction of differential expression in response to JV3 compared to all other strains

Of the 225 VSR genes, 128 are connected within the network (AUC = 0.6776, *p* = 2.81E-22) (Fig. 5; Supplemental Table 9, Additional File 3). Available alleles of several of the highest-ranking genes in this network, including *sodh-1*, *dhs-3*, *F13D12.6*, *pho-1*, *acdh-1*, *C55A6.7*, *dhs-2*, and *F08A8.4* were used for functional analysis.

Because of the small number of JSR genes, WormNet was not used to prioritize these genes for functional analysis. Many of these genes had very low overall expression. Therefore, genes were chosen if the total fragments per kilobase per million mapped reads (FPKM) for all four treatments was greater than 30. We used an available allele of the upregulated gene *nhr-110* and used CRISPR/Cas9 to generate a deletion of the downregulated gene *W02A2.8* for functional analysis (Additional File 4).

### Functional analysis of common *S. maltophilia* and strain-specific genes

Survivorship of mutants compared to *wild-type C. elegans*, quantified by Cox proportional hazards test, was used to determine whether candidate genes were important for response to treatment bacteria *E. coli* OP50 and *S. maltophilia* K279a, JCMS, and JV3. We tested the simple hypothesis that CPSR genes are important for response to both JCMS and JV3, JSR genes are important for response to JCMS, and VSR genes are important for response to JV3; therefore, mutants of these genes will result in increased or decreased susceptibility to JCMS and JV3, just JCMS, or just JV3, respectively, as compared to *wild-type*.

**Fig. 6:**
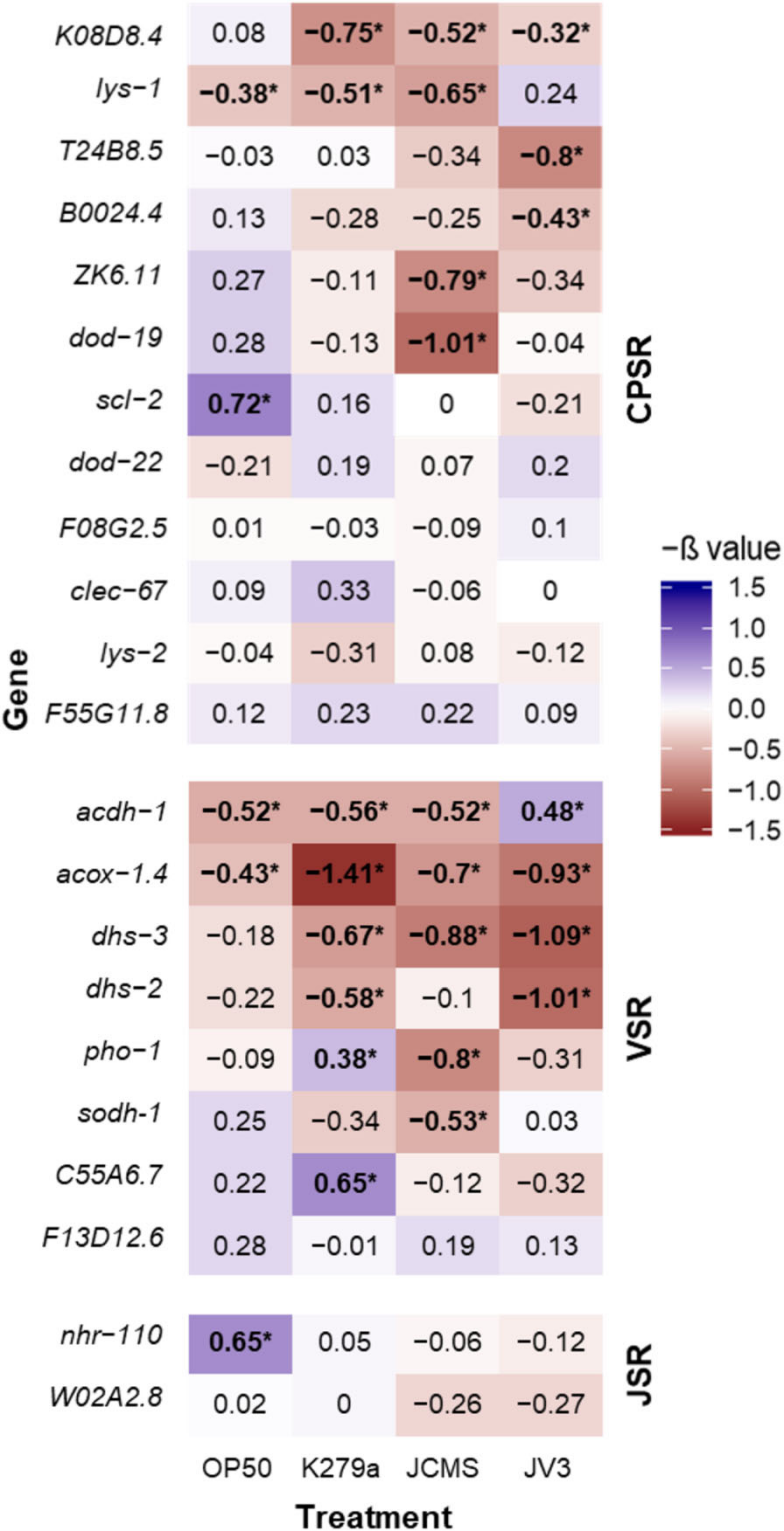
Relative survival of *C. elegans* mutants in CPSR, VSR, and JSR genes. Relative survival, determined by Cox proportional hazards mixed effects model, of mutants relative to *wild-type* on each bacterial treatment. General linear hypothesis testing was used to compare each mutant to *wild-type* on each bacterial treatment. Heatmap was made in R with ggplot2 using the -β from the Cox mixed effects model. The Benjamini-Hochberg procedure was used to adjust *p*-values for false discovery rates associated with multiple comparisons, with bold numbers and asterisks indicating significant -β values (*p*-value < 0.05). Genes are ordered based on number of bacteria in which phenotypic effects are observed

Mutations in four of the 12 CPSR candidate genes (*lys-1, K08D8.4, ZK6.11,* and *dod-19*) caused significantly increased susceptibly to JCMS, while three mutations (*B0024.4*, *K08D8.4*, and *T24B8.5*) also caused increased susceptibility to JV3 (Fig. 6; Table 3; Additional File 5). Mutations in two of these genes (*K08D8.4 and lys-1*) also increased susceptibility to K279a (Fig. 6; Table 3; Additional File 5). All of these genes, apart from *B0024.4*, were previously reported to play a role in innate immune response based on GO terms. In addition, mutations in *lys-1* caused increased susceptibility to *E. coli* OP50, while *scl-2* caused decreased susceptibility to OP50. Overall, mutations in seven of the 12 CPSR genes caused significant differences in survival in response to at least one bacterial treatment.

**Table 3:** Cox proportional hazard ratios for common *S. maltophilia* and strain-specific genes

						Relative to *wildtype*	
	Nematode	Bacteria	N	M	SE	Hazard Ratio exp(β)	*p* value
	*wildtype*	OP50	516	10.59	0.23	NA	
		K279a	615	10.87	0.19	NA	
		JCMS	608	5.44	0.07	NA	
		JV3	580	2.53	0.03	NA	
**CPSR genes**	*B0024.4*	OP50	26	10.50	0.78	0.88	0.698
	*(mh82)*	K279a	58	8.66	0.55	1.32	0.146
		JCMS	90	4.74	0.16	1.28	0.14
		JV3	58	2.06	0.07	1.54	.03*
	*F08G2.5*	OP50	46	10.43	0.77	0.99	0.988
	*(mh86)*	K279a	59	10.85	0.68	1.03	0.897
		JCMS	57	5.39	0.23	1.10	0.696
		JV3	59	2.72	0.07	0.91	0.68
	*ZK6.11*	OP50	46	12.24	0.74	0.76	0.194
	*(ok3738)*	K279a	57	10.39	0.62	1.12	0.636
		JCMS	58	4.47	0.14	2.19	1.33E-6*
		JV3	56	2.27	0.08	1.41	0.07
	*T24B8.5*	OP50	58	10.69	0.60	1.04	0.897
	*(ok3236)*	K279a	61	10.20	0.60	0.97	0.923
		JCMS	60	4.88	0.16	1.41	0.053
		JV3	57	1.94	0.07	2.23	8.9E-7*
	*dod-19*	OP50	56	11.84	0.64	0.76	0.14
	*(ok2679)*	K279a	60	9.80	0.57	1.13	0.577
		JCMS	60	4.07	0.12	0.36	7.27E-11*
		JV3	58	2.36	0.10	1.04	0.873
	*dod-22*	OP50	74	10.22	0.54	1.23	0.216
	*(ok1918)*	K279a	90	13.23	0.41	0.83	0.216
		JCMS	89	5.87	0.18	1.07	0.702
		JV3	89	0.28	0.08	0.82	0.194
	*K08D8.4*	OP50	79	10.95	0.66	0.92	0.696
	*(mh101)*	K279a	90	7.62	0.50	2.11	1.46E-8*
		JCMS	87	4.82	0.17	1.68	.000154*
		JV3	85	2.36	0.08	1.37	.038*
	*lys-1*	OP50	82	10.09	0.42	1.46	.010*
	*(ok2445)*	K279a	90	9.17	0.44	1.67	.000147*
		JCMS	88	4.76	0.14	1.92	1.33E-6*
		JV3	86	2.77	0.08	0.78	0.129
	*clec-67*	OP50	79	11.56	0.53	0.92	0.681
	*(ok2770)*	K279a	55	13.20	0.68	0.71	0.077
		JCMS	57	5.46	0.24	1.06	0.8
		JV3	53	2.67	0.10	1.00	0.988
	*lys-2*	OP50	85	10.44	0.50	1.04	0.853
	*(tm2398)*	K279a	58	10.21	0.54	1.36	0.077
		JCMS	58	5.71	0.19	0.92	0.698
		JV3	55	2.59	0.08	1.13	0.606
	*F55G11.8*	OP50	59	10.51	0.61	0.89	0.601
	*(gk3130)*	K279a	58	11.55	0.51	0.79	0.216
		JCMS	60	5.65	0.19	0.80	0.217
		JV3	57	2.44	0.10	0.92	0.698
	*scl-2*	OP50	51	15.43	0.73	0.49	2.97E-5*
	*(tm2428)*	K279a	54	11.72	0.64	0.85	0.45
		JCMS	56	5.75	0.17	1.00	0.988
		JV3	55	2.42	0.12	1.24	0.268
**VSR genes**	*acdh-1*	OP50	50	7.12	0.77	1.68	.00688*
	*(ok1489)*	K279a	59	8.53	0.49	1.74	.00178*
		JCMS	58	4.59	0.19	1.68	.00427*
		JV3	58	2.68	0.11	0.62	.008*
	*sodh-1*	OP50	53	10.55	0.64	1.09	0.698
	*(ok2799)*	K279a	54	9.48	0.56	1.41	0.065
		JCMS	56	4.71	0.15	1.7	.00164*
		JV3	55	2.5	0.11	0.97	0.897
	*pho-1*	OP50	54	12.11	0.69	0.78	0.211
	*(tm5302)*	K279a	55	12.47	0.74	0.68	.0405*
		JCMS	55	4.58	0.18	2.22	1.33E-6*
		JV3	60	2.33	0.11	1.36	0.088
	*C55A6.7*	OP50	57	11.91	0.69	0.80	0.234
	*(tm6807)*	K279a	59	13.51	0.75	0.52	4.71E-5*
		JCMS	59	5.39	0.15	1.13	0.6
		JV3	57	2.4	0.07	1.37	0.077
	*acox-1.4*	OP50	58	9.91	0.42	1.53	.0137*
	*(tm6415)*	K279a	60	6.2	0.33	4.10	<2E-16*
		JCMS	57	4.63	0.17	2.02	1.47E-5*
		JV3	55	2.03	0.08	2.53	1.38E-8*
	*dhs-3*	OP50	55	12	0.67	1.20	0.37
	*(tm6151)*	K279a	58	9.16	0.65	1.95	4.45E-5*
		JCMS	55	4.87	0.15	2.41	4.04E-8*
		JV3	55	2.04	0.08	2.97	2.31E-9*
	*F13D12.6*	OP50	53	11.92	0.64	0.75	0.146
	*(tm7051)*	K279a	58	10.98	0.51	1.01	0.216
		JCMS	56	6	0.15	0.82	0.32
		JV3	57	2.59	0.12	0.88	0.566
	*dhs-2*	OP50	27	9.3	0.80	1.24	0.474
	*(tm7516)*	K279a	53	7.72	0.43	1.78	.00095*
		JCMS	53	4.91	0.20	1.11	0.681
		JV3	53	1.64	0.09	2.74	2.35E-11*
**JSR genes**	*nhr-110*	OP50	30	14.23	0.77	0.52	.00427*
	*(gk987)*	K279a	58	9.69	0.57	0.95	0.853
		JCMS	58	5.16	0.13	1.06	0.8
		JV3	57	2.29	0.07	1.13	0.619
	*W02A2.8*	OP50	59	11.27	0.56	0.98	0.929
	*(mh87)*	K279a	59	10.42	0.65	1.00	0.988
		JCMS	59	5.22	0.17	1.29	0.15
		JV3	57	2.35	0.1	1.31	0.14

Mean survival (M), standard error of the mean (SE), and sample size (N), are given for each nematode genotype and bacterial treatment combination. *Wild-type* statistics were determined from combining all *wild-type* data from all experiments. Hazard ratios (natural log(β)) indicate the treatment hazard divided by the hazard of *wild-type* (first column) across all experiments. The hazard is defined as the probability of a nematode dying at a given time. Hazard ratios and associated FDR adjusted p-values for each comparison were determined using Cox proportional hazards mixed effects model and general linear hypothesis tests and applying the Benjamini-Hochberg procedure to adjust for multiple comparisons in R. Asterisk indicate significant p-values (*p*<0.05)

**Fig. 7:**
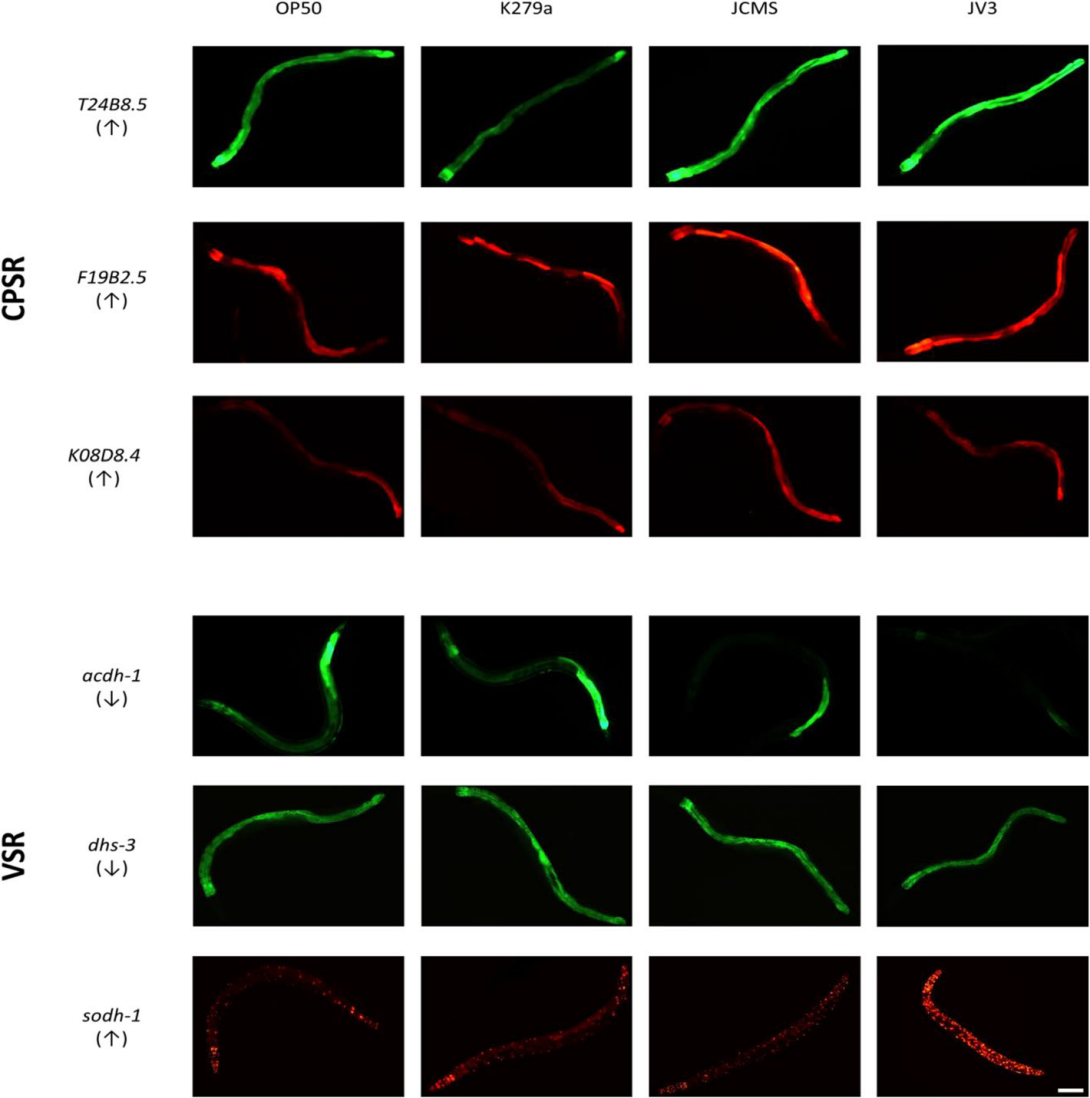
A majority of tested CPSR and VSR genes are expressed in innate immune response tissues. Expression of several CPSR (*T24B8.5, F19B2.5*, *K08D8.4*) and VSR (*sodh-1, acdh-1, dhs-3*) genes using transcriptional or translational fluorescent protein fusions upon exposure to *S. maltophilia* or *E. coli* OP50. Up- or down- arrows below each gene name indicate direction of expression in reference to the pathogenic strains (up/down-regulated in response to JV3 and/or JCMS). All transgenes are primarily expressed in the intestine, except for *sodh-1*, which is also expressed in several other tissues. Columns show expression of worms exposed to *E. coli* OP50 and *S. maltophilia* K279a, JCMS, or JV3 for 24 h at 100x magnification. The images chosen represent mean average intensity across 10–18 worms (see Additional File 8). Scale bar indicates 100 μm, anterior is to the left, and ventral is up for all pictures. Note that expression in the AIY interneuron in the *T24B8.5* transgenic strain is due to a *ttx-3*:GFP marker and not *T24B8.5* expression and the *sodh-1* expression construct contains a nuclear localization signal

Mutations in three of the eight VSR candidate genes (*acox-1.4*, *dhs-3*, *dhs-2*) caused significantly decreased survival in response to JV3, while mutations in *acdh-1* resulted in increased lifespan (Fig. 6; Table 3; Additional File 6). However, worms with mutations in all four of these genes also result in significant differences in survival in response to at least one other bacterial strain tested, suggesting that although these genes are specifically differentially expressed in response to JV3, they are also important for survival under other conditions. Additionally, mutations in *C55A6.7* and *pho-1* decreased susceptibility to K279a, and mutations in *pho-1* and *sodh-1* increased susceptibility to JCMS (Fig. 6; Table 3; Additional File 6). Overall, seven of the eight VSR genes are important for the response to at least one *S. maltophilia* strain. Interestingly, while only two of the eight genes are involved in innate immune response based on GO terms (*C55A6.7* and *acdh-1*), seven of the eight genes have GO terms associated with metabolic processes, including oxidation-reduction (*acdh-1, acox-1.4, dhs-3, dhs-2, sodh-1*), proteolysis (*F13D12.6*), and dephosphorylation (*pho-1*). In addition, all of these genes, except *dhs-2* and *C55A6.7,* have been shown to be expressed in the intestine [36–39] (Fig. 7), the site of *S. maltophilia* accumulation and proposed pathogenesis [12]. Therefore, although these genes do not seem to be important exclusively for JV3 survival, they do seem to be important for survival in response to *S. maltophilia* overall. Mutations in *acdh-1* and *acox-1.4* also increased susceptibility to *E. coli* OP50, possibly suggesting a more general role in *C. elegans* survival.

Only two JSR genes were functionally analyzed, *nhr-110* and *W02A2.8*. *nhr-110* mutants were significantly less susceptible to OP50, but no differences were seen in survival in response to JCMS or other *S. maltophilia* strains (Fig. 6; Table 3; Additional File 7).

In addition to survival analyses, we visualized the expression patterns for several CPSR and VSR genes. Several transgenic strains were available from stock centers, including transcriptional reporters for *T24B8.5*, *acdh-1, sodh-1*, and a translational reporter for *dhs-3*. We also generated translational reporters for *K08D8.4* and *F19B2.5*. Intensity and location of expression were measured in response to all *S. maltophilia* strains and *E. coli* OP50 at 12 and 24 h of exposure. Interestingly, at 12 h, except for *T24B8.5,* expression patterns did not reflect the RNA-sequencing results (Additional File 8). However, at 24 h, expression profiles of all expression constructs across treatments correlated with the RNA-sequencing results (Fig. 7; Additional File 8). This delay in observed differential expression of reporter constructs could be due to multiple factors, including folding and processing of fluorescent proteins, potential degradation in down regulated tissues or accumulation of visible amounts of fusion proteins. However, overall, these observed patterns validate our transcriptomic results.

All of these genes were expressed in the intestine, but localization of expression was also seen in the hypodermis (*sodh-1* and *acdh-1*), muscle (*sodh-1*), nervous system (*sodh-1*), and head (*K08D8.4, sodh-1,* and *acdh-1*) (Additional File 9). The intestine and hypodermis are common sites of pathogen infection, whereas the nervous system has also been shown to play a role in pathogen recognition and immune response in *C. elegans* [40–43]. Therefore, expression of differentially expressed genes in response to *S. maltophilia* correlates with common tissues involved in innate immune response.

## Discussion

### Patterns of gene expression in response to different levels of *S. maltophilia* virulence

This study utilized a transcriptomic approach to identify genetic responses to *S. maltophilia* strains of differing pathogenicity. Our previous study that identified differentially expressed genes in response to *E. coli* OP50, *S. maltophilia* K279a, and *S. maltophilia* JCMS after 24 h found almost all genes were differentially expressed between nonpathogenic K279a and pathogenic JCMS, suggesting that the nematode response differs only to the presence of virulence. This study further examines these findings by adding an even more pathogenic strain, *S. maltophilia* JV3, to determine whether there are strain similarities and differences based on level of virulence. In this case, we identified differences in the nematode response between species (*E. coli* and *S. maltophilia*) and levels of virulence (Figs. 2, and 3). However, the expression patterns between the nonpathogenic strains are more similar than those of the pathogenic strains (Fig. 2), suggesting that although there are differences between species, the majority of gene expression changes are based on the presence of virulence.

To determine the existence of a common pathogenic *S. maltophilia* response (CPSR), we identified 145 genes that were differentially expressed between all pathogenic and non-pathogenic comparisons (Fig. 3; Supplemental Table 3, Additional File 1). A majority of these genes (89%) were upregulated in response to JCMS and JV3 (Supplemental Table 3, Additional File 1). This is consistent with previous transcriptomic patterns of genes differentially expressed in *C. elegans* upon pathogen exposure, in which a majority of genes were upregulated in response to a variety of bacterial and fungal pathogens [23, 24, 44]. However, another study that compared transcriptomic responses to a variety of bacterial pathogens found that a majority of genes were down-regulated in response to Gram-negative bacteria and up-regulated in response to Gram-positive bacteria [45]. Furthermore, our previous study of the response of *C. elegans* to *S. maltophilia* JCMS found a majority of genes were down-regulated [25]. However, that study used microarrays to detect expression differences induced at 24 h of exposure, whereas here we used RNA sequencing at 12 h of exposure. Therefore, this directionality of gene expression could be strain- or time-specific. In fact, 200 of the 225 (89%) of the genes specifically differentially expressed in response to JV3 (VSR genes) are downregulated (Supplemental Table 5, Additional File 1). This supports the idea that directionality of gene expression in *C. elegans* may be strain specific, and different virulence mechanisms or host responses may play a role in these patterns.

In comparison to VSR genes (225), there are very few genes (14) specifically differentially expressed in response to JCMS (JSR) (Supplemental Table 4, Additional File 1) and most of these genes were expressed at low levels. Overall, this suggests that at 12 h of exposure JCMS does not employ unique virulence mechanisms as it appears JV3 does and therefore does not lead to unique host responses.

### The CPSR and VSR genes identify functions that play different roles in the response to *S. maltophilia*

We used GO enrichment and functional analysis of mutations to begin to understand functional roles of the CPSR genes. GO enrichment analysis identified processes involved in defense response, particularly response to Gram-negative bacteria, as well as a molecular function in carbohydrate binding and the cellular component of membrane raft (Table 1). Genes with the GO term “molecular function of carbohydrate binding” all belong to *clec* or *lec* family, which are structurally similar to carbohydrate binding proteins. Although not all nematode *clec* and *lec* genes encode molecules that bind carbohydrates, many are predicted to be secreted proteins that may act as immune effectors [34, 46]. Many *clec* genes are expressed in the *C. elegans* intestine and are differentially expressed in response to pathogens; a recent review determined that 237 of 283 *clec* genes are differentially expressed during pathogen infection [46]. We analyzed a mutation in only one *clec* gene, *clec-67*, and no differences were found in survival between the *clec-67* mutant and *wild-type*. This might be explained by functional redundancy among the *clec* family proteins, as many of these proteins are structurally similar. In fact, there are nine other *clec* genes among the CPSR genes, three of which (*clec-70*, *clec-83*, and *clec-85*) are paralogous to *clec-67*, suggesting similar functional roles. Therefore, even though these genes may play a role in innate immune response, mutations in one *clec* gene alone may not result in an effect on survival upon pathogen-challenge. Future work to interfere with the functions of multiple *clec* genes at one time will shed light on this possibility.

Membrane rafts, or lipid rafts, are membrane domains that contain high concentrations of cholesterol and glycosphingolipids [47]. Membrane rafts also serve as sites of colocalization between membrane proteins and signaling pathway components, such as components of MAPK and insulin-like signaling pathways, both of which are known to play roles in innate immunity and defense in *C. elegans* [19–21, 47, 48]. In addition, many of the CPSR membrane raft genes also have biological processes associated with the innate immune response. To further support this, mutations in three paralogous membrane raft genes, *B0024.4*, *ZK6.11*, and *dod-19*, caused increased susceptibility to either JCMS or JV3. Mutations in two other genes, *T24B8.5* and *lys-1*, were also shown to increase susceptibility to at least one *S. maltophilia* strain. *T24B8.5* is regulated by the PMK-1 pathway [49], and *lys-1* is regulated by the DBL-1 pathway [22], both well-known innate immune pathways. Therefore, it is possible that these membrane raft proteins are involved in innate immune signaling pathways, where increasing expression of the membrane raft proteins results in increased signaling and therefore increased expression of innate immune effector genes. Further analysis of these membrane raft genes and their potential role in innate immune signaling pathways could reveal novel gene functions that are important components in the response to *S. maltophilia* and potentially other pathogens.

On the other hand, GO enrichment analysis of VSR genes identified many processes and functions involved in metabolism (Table 2). Genes with GO terms “flavonoid glucuronidation”, “flavonoid biosynthetic process”, and “UPD-glycosyltransferase” consist almost entirely of glycosyltransferase family proteins, a large protein family in *C. elegans*, comprising 265 genes [50]. However, a direct linkage between glycosyltransferases and innate immunity has not yet been observed. Genes with GO term “oxidation-reduction process” include many dehydrogenase and oxidase enzymes. Interestingly, mitochondria, the location of many dehydrogenases, have been shown to be involved in pathogen recognition [51]. Specifically, FADH_2_-dependent dehydrogenase activity in mice macrophages increases upon exposure to *E. coli* [52]. Furthermore, GO terms associated with metabolic processes have previously been found to be enriched among down-regulated genes in response to *S. maltophilia* and *B. thuringiensis* [25, 27]. One explanation might be that some pathogens, including *S. maltophilia* JV3, utilize mechanisms to interfere with metabolism in *C. elegans*, resulting in their own increased virulence. On the other hand, this downregulation may be a defense mechanism used by *C. elegans* to decrease metabolites needed for pathogen survival or pathogenesis. Five genes in this category, *dhs-2*, *dhs-3*, *acdh-1*, *sodh-1*, and *acox-1.4*, were chosen for functional analysis, and mutations in all had an effect on survival in response to at least one *S. maltophilia* strain. The involvement of VSR genes in general metabolism could explain their more general effect on survival (Fig. 6). However, further analyses of these genes in *C. elegans* are needed to fully understand their role in response to *S. maltophilia* JV3 and other pathogens. These analyses, along with JV3 genome sequence analysis, could provide insight into unique virulence mechanisms employed by JV3.

In addition to strain-specific gene expression patterns, we also observe strain-specificity in our functional analyses (Fig. 6). For example, while mutations in the VSR gene *acdh-1* increase susceptibility to OP50, K279a, and JCMS, the same mutation causes a significant decrease in susceptibility to JV3. A similar pattern is observed with mutations in the CPSR gene *lys-1* that also cause increased susceptibility to OP50, K279a, and JCMS, but non-significantly decreased susceptibility to JV3. This further supports our hypothesis that JV3 utilizes unique mechanisms that result in strain-specific genetic responses in *C. elegans*. Therefore, further understanding the function of *lys-1* and *acdh-1* and their role in response to JV3 may uncover information about its virulence mechanisms.

### Host responses could exist along a continuum of response to increased pathogenicity

Overall, data from the survival analyses do not support the simple hypothesis that CPSR genes are necessary for survival on JCMS and JV3, while JSR and VSR genes are necessary for survival on only JCMS or JV3, respectively, as mutations in a majority of genes did not affect survival of *C. elegans* in that strain-specific manner (Fig. 6; Table 3). However, it appears that overall, these genes do play a role in response to *S. maltophilia*, as 13 of 22 candidate CPSR, JSR, and VSR gene mutants display significant differences in survival upon exposure to at least one *S. maltophilia* strain. Furthermore, mutations in many of the genes we analyzed caused significant differences in survival upon exposure to K279a in addition to the pathogenic strains, JCMS and JV3 (Fig. 6; Table 3). Genome sequencing of *S. maltophilia* K279a has identified a variety of virulence factors [53], suggesting that it may in fact have pathogenic potential. Whereas wild-type *C. elegans* may be able to defend against K279a infection, mutations in innate immune and defense genes may cause *C. elegans* to become more susceptible to K279a. Therefore, identifying and functionally analyzing differentially expressed genes in *C. elegans* in response to all *S. maltophilia* strains could also uncover important genetic components of the response to *S. maltophilia* virulence.

Although RNA sequencing examines gene expression on a genome-wide scale, it only captures expression profiles at one point in time. Therefore, it is possible that the interaction between *C. elegans* and the different *S. maltophilia* strains is more nuanced than predicted by the simple hypothesis that genes differentially expressed at one point in time will affect nematode survival. For example, because JV3 is more virulent than JCMS and K279a, the JV3-exposed worms could be at a more advanced stage of pathogenesis than the JCMS and K279a-exposed worms at 12 h. Thus, in addition to any strain specific effects, differential responses at a single time point might also reflect different stages of pathogenesis. This idea is supported by our observation that among the upregulated CPSR genes, most were further upregulated in response to JV3 as compared to JCMS (Supplemental Table 3, Additional File 1). Survival analysis, unlike RNA-sequencing, can provide information about gene function across life history. The observation that mutations in many genes differentially expressed in response to specific *S. maltophilia* strains affect survival upon exposure to multiple strains of *S. maltophilia* suggests that these genes may be important for response to *S. maltophilia* more generally. That these mutations can cause varying effects on survival in response to different strains also indicates complexities of gene function within this general response. Transcriptional analysis of the *C. elegans* response to different strains across time of exposure could help determine the relative importance of time-specific and strain-specific responses.

## Conclusion

Using transcriptomic data to understand and analyze responses to pathogens can provide insight into overall response patterns and pathogen virulence mechanisms. Utilizing both transcriptional and functional analyses, this study illustrates the previously supported idea that there are common signatures of pathogen infection in *C. elegans*, but also unique species and even strain specific responses. More specifically, we provide evidence to support that different strains of *S. maltophilia* of varying pathogenicity cause different transcriptomic signatures, and JV3 elicits a unique downregulation of metabolic genes in *C. elegans* that are involved in survival more generally. Therefore, to fully understand virulence of bacteria and pathogenesis in *C. elegans* for both clinical and environmental applications, a variety of bacterial species and strains need to be investigated. Because *S. maltophilia* is a human opportunistic pathogen, understanding virulence mechanisms of and host responses to a variety of *S. maltophilia* strains could lead to novel information about *S. maltophilia* infection. Finally, focusing on more natural host-pathogen interactions promises to provide a more realistic understanding of host responses.

## Methods

### Nematode and bacteria strains and growth

The following *C. elegans* strains were obtained from the CGC: RB1573 *dod-22(ok1918),* VC1749 *F55G11.8(gk3130) ZK185.2(gk828),* VC3059 *ZK6.11(ok3738),* VC2477 *T24B8.5(ok3236),* RB1893 *lys-1(ok2445),* VC2249 *dod-19(ok2679),* RB2095 *clec-67(ok2770),* VC2176 *nhr-110 (gk987),* RB2114 *sodh-1 (ok2799),* VC1011 *acdh-1(ok1489),* LIU1 [*dhs-3p::dhs-3::*GFP *+ unc-76(+)*]*,* AU78 [*T24B8.5p::*GFP*::unc-54-*3′ UTR *+ ttx-3p::*GFP*::unc-54-*3′ UTR]*,* CF2124 [*sodh-1p::*RFP (NLS) *+ rol-6(su1006)*]*,* VL717 [*acdh-1p::*GFP]*. C. elegans* strains containing the following alleles were obtained from the National BioResource Project (NBRP): *lys-2(tm2398), scl-2(tm2428), dhs-3(tm6151), F13D12.6(tm7051*), *pho-1(tm5302)*, *C55A6.7*(*tm6807*), *dhs-2(tm7516), acox-1.4(tm6415).* All alleles were outcrossed 4 times and were screened by PCR after each outcross to obtain homozygous mutants. Forward and reverse primers used to test for each allele can be found on Wormbase. Each of the mutations used in this study have molecular lesions that indicate loss of gene function. Specific details of each mutation and its molecular lesion can be found on Wormbase. Bristol *N2* strain was also obtained from the CGC and used as *wild-type*. All strains were maintained on nematode growth media (NGM) plates seeded with *E. coli* OP50 at 20 °C.

*C. elegans* strains containing the following expression constructs and alleles were generated as described below: *mhEx284*[*F19B2.5p::F19B2.5:*:wrmScarlet::*unc-54*-3′ UTR] and *mhEx283*[*K08D8.4p::K08D8.4*::wrmScarlet::*unc-54*-3′ UTR], *F08G2.5*(*mh86*), *K08D8.4(mh76), B0024.4(mh82),W02A2.8(mh87)*.

Bacterial strains include *E. coli* OP50 from the CGC, *Stenotrophomonas maltophilia* JCMS isolated by our lab in association with nematodes from Konza Prairie near Manhattan, KS [12], *Stenotrophomonas maltophilia* K279a from R. Ryan (University College Cork), *Stenotrophomonas maltophilia* JV3 from J. Tiedje (Michigan State University).

All bacteria strains were frozen at − 80 °C upon arrival and thawed frequently for experimentation. *S. maltophilia* strains are naturally Ampicillin resistant, thus were grown on Luria Broth (LB) agar containing 100 μg/mL Ampicillin to selectively isolate and maintain each strain while avoiding contamination. *E. coli* OP50 was grown on regular LB agar. Plates were incubated at 37 °C overnight and kept at 4 °C thereafter. *S. maltophilia* strains were grown in liquid LB containing 100 μg/mL Ampicillin, and *E. coli* OP50 was grown in liquid LB and shaken overnight at 37 °C. Liquid cultures were then seeded onto NGM and grown at room temperature overnight before being used for experimentation.

### RNA isolation

Wild-type nematodes were synchronized by bleaching, plated on *E. coli* OP50, and maintained at 20 °C. Synchronized larval stage 4 (L4) worms were washed several times in M9 buffer and transferred to treatment bacteria or *E. coli* OP50. Treatments included *S. maltophilia* strains K279a, JCMS, and JV3. After 12 h of exposure to treatment bacteria at 25 °C, worms were collected in M9 buffer and lysed in TRIzol® (Life Technologies). 12 h of exposure to treatments was chosen because at this point bacterial accumulation in the intestine has begun [12], but almost all worms in each treatment were still alive. Only non-contaminated, un-starved populations were used for RNA extraction, and three biological replicates were collected for each treatment. Bulk RNA was extracted from these populations using PureLink RNA Mini Kit (Invitrogen), and DNase treated using On-Column PureLink® DNase Treatment (Invitrogen) following the manufacturer’s protocol. RNA quality was checked by determining 260/280 and 260/230 absorbance ratios using a NanoDrop™ 8000 Spectrophotometer and observation of 18S and 28S rRNA bands using gel electrophoresis.

### RNA sequencing and analysis

Extracted RNA was sent to the University of Kansas Center for Molecular Analysis of Disease Pathways Genome Sequencing core facility for library preparation and sequencing. Three biological replicates, consisting of pooled bulk nematode RNA, and two technical replicates of each biological replicate were sequenced for each treatment. Libraries were sequenced on Illumina HiSeq 2500 platform resulting in 100 base pair single-end reads. Sequence quality was assessed using FastQC.

Tophat 2, which uses the short-read mapping program Bowtie [54], was used to map reads to the *C. elegans* genome. Technical replicates were combined at this step. Transcriptome and genome versions WS235 were used as the reference. Minimum intron length was set to 15 base pairs (*−i 15*) and the parameter for *-no-novel-juncs* was used. The remainder of settings were set to default. Cuffdiff, a program within Cufflinks, is used to compare expression of transcripts at the isoform-level between treatments, accounting for variability within biological replicates [55]. The parameter *-multi-read-correct* was used to account for reads mapping to multiple locations, with the remainder of settings set to default. Transcripts were considered significantly differentially expressed between treatments if the fold change > 2 and the false discovery rate (FDR)-adjusted *p*-value < 0.05. Heatmap analysis and comparison of differentially expressed genes between different conditions were performed in R (Vienna, Austria: R Foundation for Statistical Computing) using the heatmap.2 function in the package gplots. Fragments per kilobase per million (FPKM) values for each gene and treatment were log transformed, and gene expression values were centered and scaled to have mean zero and standard deviation one across the row.

### Gene ontology enrichment analysis

Differentially expressed genes of interest were queried for gene ontology (GO) term enrichment using DAVID Bioinformatics Resources 6.8 [29, 30] with the background set to the entire *C. elegans* gene list. Each gene is assigned one or more GO terms and categorized into Biological Process, Molecular Function, and Cellular Component. Gene sets of interest were analyzed using the functional annotation chart and the “ALL” database in DAVID for each GO category. Significant enrichment of GO terms was determined using a Fisher’s exact test [30]; this test associates a *p*-value, or EASE score, to each GO term based on the number of genes associated with that term as compared to background [30]. False-discovery rate (FDR) correction of EASE scores, as implemented by DAVID, was used to control for multiple testing. GO terms were considered to be significantly enriched if FDR adjusted *p*-value < 0.05.

### Mutant generation using CRISPR/Cas9

CRISPR/Cas9 was used to generate mutations in *W02A2.8*, *F08G2.5*, *K08D8.4,* and *B0024.4*. Guide RNA (gRNA) sequences were chosen within the coding sequence of the gene of interest (GOI) using the CRISPRseek package in R to select guides with high efficacy, and CRISPR design (https://crispr.mit.edu) to identify possible off-target effects. Two to four gRNAs were identified and constructed for each GOI (Additional File 10). Double-stranded gRNA sequences consisted of 20 base pairs prior to the PAM site (NGG) plus overhanging base pairs on each end that overlapped with *Bsa*I-cut pRB1017 plasmid. This overlap allowed for proper ligation of the gRNA sequence into *Bsa*I-cut pRB1017 [56].

A co-CRISPR method, described in Arribere et al., was used to facilitate detection of gene-editing events. Briefly, an injection mix of 50 ng/μl *Peft:Cas9* vector [57], 20–25 ng/μl of *dpy-10* gRNA [56], and 20–25 ng/μl of each target gRNA-carrying plasmid were injected into young adult worm gonads [58]. F_1_ Dpy worms were then moved to new plates and allowed to lay eggs. DNA was then isolated from F_1_ Dpy worms and amplified with primers targeting genomic sites flanking the gRNAs of the GOI (Additional File 10).

Gene-editing events were identified by differences in amplicon size as compared to *wild-type*, indicating an insertion or deletion in the gene. Worms containing mutant alleles were then sequenced to determine the molecular lesion and outcrossed twice to *wild-type* males to eliminate possible off-target mutations. None of the mutations we generated resulted in a visible phenotypic effect. The deletion in *W02A2.8* removes the start codon of the gene, whereas mutations in *F08G2.5*, *K08D8.4,* and *B0024.4* result in a frameshift of the coding sequence. These molecular lesions suggest loss-of-function mutations. A summary of CRISPR/Cas9 generated alleles is shown in Additional File 4.

### Generation of expression construct strains

NEBuilder HiFi DNA Assembly (New England BioLabs) was used to assemble the vector backbone (pPD95.75), promoter and gene of interest (GOI), and fluorescent tag (wrmScarlet). DNA vectors are assembled by ligating fragments with overlapping sequence using an endonuclease to create single-stranded overhangs within the overlap sequences and ligase to ligate the fragments together. In this case, the three fragments were generated via PCR using high fidelity Phusion DNA Polymerase (Thermo Fisher Scientific). Fragment 1, encoding the fluorescent protein wrmScarlet, was amplified from pSEM89_*egl-23::SL2::wrmScarlet* [59] using forward primer 5′- ATGGTCAGCAAGGGAGAGGCAG − 3′ and reverse primer 5′- TTACTTGTAGAGCTCGTCCATTCCTCC − 3′. Fragment 2, the plasmid pPD95.75, which contains GFP followed by the *unc-54* 3’UTR, was amplified using forward primer 5′- GACGAGCTCTACAAGTAACATTCGTAGAATTCCAACTGAGCG − 3′ and reverse primer 5′- TTTTTCTACCGGTACCCTCCAAGGG − 3′. This generated a linearized vector backbone that included a majority of the plasmid, excluding the GFP coding sequence. Fragment 3, which contains the GOI driven by its endogenous promoter (either 2 kb upstream of the gene or to the nearest upstream gene) and differed for each gene, was amplified with the following primers: F19B2.5 driven by the F19B2.5 promoter (pF19B2.5::F19B2.5) forward primer 5′- GGAGGGTACCGGTAGAAAAATGATTATTTCCGGCTCGGG - 3′ and reverse primer 5′- CTCCCTTGCTGACCATCTGGCTGTCGTCGGCTC - 3′, and K08D8.4 driven by the K08D8.4 promoter (pK08D8.4::K08D8.4) forward primer 5′- GAGGGTACCGGTAGAAAAACACCCAAGGATTTGAAG − 3′ and reverse primer 5′- CTCTCCCTTGCTGACCATGACCAGCATAACAAAACC − 3′. The primers used to amplify the vector backbone and the promoter/GOI fragment contain the appropriate overlap sequence, resulting in circular assembly of the promoter and GOI fragment ligated to the wrmScarlet fragment ligated to the vector backbone. Fragments were then gel purified using PureLink™ Quick Gel Extraction Kit (Invitrogen), followed by assembly and cloning using NEBuilder HiFi Assembly Master Mix and Cloning Kit following manufacturer’s protocol.

Colonies containing possible positive constructs after cloning were tested by PCR to ensure the fragments were assembled correctly. DNA was extracted from confirmed correct colonies. Finally, 20–50 ng/ul of each construct along with 20 ng/ul *dpy-10*(+) plasmid were injected into Dpy worms. F_2_*wild-type* worms were then screened for wrmScarlet expression, and 3 independent transgene-containing lines were obtained for each GOI, with one representative line chosen for further analysis.

### Gene expression analysis

Nematodes containing extrachromosomal or integrated alleles for transcriptional or translational fluorescent protein fusions were moved to treatment bacteria (OP50, K279a, JCMS, or JV3) at the L4 stage. After 12 and 24 h of exposure to treatment bacteria, nematodes were anesthetized (10 mM sodium azide) for observation at 100x magnification using a Leica DM6 microscope equipped with epifluorescence and differential interference contrast (DIC) optics. To quantify expression, 10–18 nematodes expressing each construct were imaged per treatment. We quantified expression within the intestine, the main immune organ of the worm, by marking the region posterior of the pharynx to the posterior intestine as a region of interest (ROI) and quantifying average fluorescent intensity (pixel sum within region / area) using LAX software (Leica). Mean average intensities were compared across treatments at each time point (12 and 24 h) using a Tukey HSD multiple comparison test in R. Images were also taken of young adult worms containing expression constructs exposed to OP50 at 400x magnification to show localization of expression.

### *C. elegans* survival assays

Treatment bacteria were cultured in liquid LB (with Ampicillin for *S. maltophilia* strains) overnight and 75 μl of bacteria were plated onto NGM agar plates the day prior to use. Worms were bleached to synchronize and reared at 20 °C on lawns of *E. coli* OP50. For survival assays, 10–12 L4 worms were transferred to each treatment plate, with three replicates of each treatment, and maintained at 25 °C. Worms were transferred to new plates every day until they stopped laying eggs to separate them from their progeny. Surviving worms were recorded each day and dead worms were removed from plates, as determined by lack of movement following prodding with a platinum wire pick. Plates that became contaminated or worms that crawled off the agar and died were removed from data analysis.

Statistical analyses were performed in R to determine differences between the independent variable nematode genotype, with the dependent variable being the probability of nematode death on a given day. Survival probability estimates over time were determined using the Kaplan-Meier formula using the survival package in R. The Cox proportional hazards mixed effects model was then used to compare the effects of nematode genotype using the coxme package in R. *Wild-type* worms were included in every round of experimentation, and during analysis, mutant nematode strains were compared to *wild-type* nematodes from all experimental rounds. The experimental rounds were treated as random variables and differences between rounds were accounted for within the model.

General linear hypothesis tested was performed using the multcomp package in R to compare mutant nematode strains to *wild-type* nematodes on each bacterial treatment. The Benjamini-Hochberg procedure was used to adjust *p*-values for false discovery rates associated with multiple comparisons, with adjusted *p*-values < 0.05 considered to be significant. The relative survival heatmap was made in R with ggplot2 using the –(β) value for each comparison to *wild-type*.

## Supplementary information

**Additional file 1 **Differentially expressed genes between treatment comparisons. List of all differentially expressed genes between comparisons. Genes were considered differentially expressed if fold-change > 2 and FDR-adjusted *p*-value < 0.05. Fragments per kilobase per million (FPKM) values are shown for each treatment. Additional sheets contain CPSR, VSR, and JSR genes with FPKM values and up/down-regulation in reference to the pathogenic strains (up/down-regulated in response to JV3 and/or JCMS). Asterisk indicates different direction of regulation depending on the comparison considered.

**Additional file 2.** GO analysis of upregulated CPSR and downregulated VSR genes. Gene ontology (GO) enrichment analysis was performed on the upregulated CPSR genes or the downregulated VSR genes using DAVID Bioinformatics Resources 6.8. GO analysis identifies terms relating to the biological process, molecular function, or cellular component that are significantly enriched among a list of genes. Indented terms indicate child terms, or subcategories, of the term listed above, with the parent term left-aligned. Note that the degree of indention of each term does not reflect absolute GO term level within each category. Count is the number of genes corresponding to each GO term. Percent is the count / total considered in analysis. FDR is the false discovery rate-corrected EASE enrichment score to account for multiple testing. Only terms with FDR < 0.05 and the most descriptive term for each unique gene list are shown.

**Additional file 3.** CPSR and VSR genes ordered based on gene network rank. WormNet v2 was queried with the 145 CPSR genes or 225 VSR genes. Connected genes are ordered based on WormNet score, which is based on the number of connections that gene has and the strength of the evidence for those connections (Score). C = number of connected CPSR genes to the listed gene. Up/down-regulated = direction of differential expression in response to pathogenic compared to nonpathogenic strains. Asterisk indicates different direction of regulation depending on the comparison considered.

**Additional file 4 **CRISPR/Cas9 generated alleles. Genes for which CRISPR/Cas9 alleles were generated are shown. Exons are indicated by green boxes and introns are indicated by black lines. Gene sizes are not to scale, but exon/intron size within genes is to scale. Relative location of the gRNAs is indicated by circles above the gene, and location of mutation is indicated in red (lines for deletions, triangles for insertions). All isoforms of *K08D8.4* and *W02A2.8* are shown. *K08D8.4* mutations are predicted to result in loss of function of all isoforms; *W02A2.8c* may be expressed but is not differentially expressed between treatments. Mutation sequence and flanking sequence is shown on the right, with mutation sequence shown in red font (number in parentheses represents size of deletion).

**Additional file 5 **Mutations in CPSR genes result in a variety of survival patterns upon *S. maltophilia* exposure. Survivorship of *wild-type* nematodes and CPSR mutants on *S. maltophilia* JCMS, K279a, JV3, and *E. coli* OP50. Survival estimates were determined using Kaplan-Meier estimates generated in R. For these experiments, 10–12 worms were synchronized, picked onto each treatment bacterial lawn (3 plates per treatment/nematode combination) and the number of living worms was recorded daily. 2–3 replicates were completed for all bacterial and *C. elegans* strain combinations. Sample sizes, hazard ratios and *p*-values generated form Cox proportional hazards tests are shown in Table 3.

**Additional file 6 **Mutations in VSR genes result in a variety of survival patterns upon *S. maltophilia* exposure. Survivorship of *wild-type* nematodes and VSR mutants on *S. maltophilia* JCMS, K279a, JV3, and *E. coli* OP50. Survival estimates were determined using Kaplan-Meier estimates generated in R. For these experiments, 10–12 worms were synchronized, picked onto each treatment bacterial lawn (3 plates per treatment/nematode combination) and the number of living worms was recorded daily. 2–3 replicates were completed for all bacterial and *C. elegans* strain combinations. Sample sizes, hazard ratios and *p*-values generated form Cox proportional hazards tests are shown in Table 3.

**Additional file 7 **Mutations in JSR genes result in a variety of survival patterns upon *S. maltophilia* exposure. Survivorship of *wild-type* nematodes and JSR mutants on *S. maltophilia* JCMS, K279a, JV3, and *E. coli* OP50. Survival estimates were determined using Kaplan-Meier estimates generated in R. For these experiments, 10–12 worms were synchronized, picked onto each treatment bacterial lawn (3 plates per treatment/nematode combination) and the number of living worms was recorded daily. 2–3 replicates were completed for all bacterial and *C. elegans* strain combinations. Sample sizes, hazard ratios and *p*-values generated form Cox proportional hazards tests are shown in Table 3.

**Additional file 8 **Expression construct quantification at 12 and 24 h. Expression levels of several CPSR (*T24B8.5, F19B2.5*, *K08D8.4*) and VSR (*sodh-1, acdh-1, dhs-3*) genes using transcriptional or translational fluorescent protein fusions upon exposure to *S. maltophilia* or *E. coli* OP50. L4 worms containing expression constructs were moved to *S. maltophilia* K279a, JCMS, JV3, or *E. coli* OP50. After 12 and 24 h, images were taken of 10–18 worms for each treatment and expression construct, and average intensity was measured for each worm. Plots show mean average intensity and standard error for each time, treatment, and expression construct. Letters indicate significant differences across treatments for each time point (Tukey’s HSD).

**Additional file 9 **Expression construct localization. Expression of several CPSR (*T24B8.5, F19B2.5*, *K08D8.4*) and VSR (*sodh-1, acdh-1, dhs-3*) genes using transcriptional or translational fluorescent protein fusions upon exposure to *E. coli* OP50 at 400x in young adult worms. *T24B8.5, F19B2.5* and *dhs-3* are only expressed in the intestine, so the anterior intestine is shown. *K08D8.4*, *acdh-1* and *sodh-1* are also expressed in the head, which is shown. Brackets indicate head and intestine region on each worm. Unclosed brackets signify that region extends out of frame. Scale bar indicates 20 μm, anterior is to the left, and ventral is up for all pictures. Note that expression in the AIY interneuron (arrow) in the *T24B8.5* transgenic strain is due to a *ttx-3*:GFP marker and not *T24B8.5* expression and the *sodh-1* expression construct contains a nuclear localization signal.

**Additional file 10 **CRISPR/Cas9 target gene primers. Forward and reverse primers for each gRNA were annealed and ligated into pRB1017. gRNA sequence (20 bp prior to the PAM site) are underlined for each primer, non-underlined bases are included in the primer sequence for proper ligation into *Bsa*I digested pRB1017. Primers flanking the gRNA target loci were used to amplify DNA from candidate mutant worms to detect insertions or deletions based on amplicon size. Odd numbers of flanking primers listed for any gene were tested in combinations (forward primer was tested with each reverse primers).

## Data Availability

The datasets generated and/or analyzed during the current study are available in the NCBI Sequence Read Archive (SRA) repository under BioProject ID PRJNA561173.

## Abbreviations

FDR: False discovery rate
CPSR: Common pathogenic *S. maltophilia* response
JSR: JCMS-specific response
VSR: JV3-specific response
GO: Gene ontology
DAVID: Database for Annotation, Visualization, and Integrated Discovery
AUC: Area under curve
ROC: Receiver operating characteristic
FPKM: Fragment per kilobase per million
CRISPR: Clustered regularly interspersed short palindromic repeats
