# Supplementary material for: Identification and characterization of differentially expressed genes in Caenorhabditis elegans in response to pathogenic and nonpathogenic Stenotrophomonas maltophilia
Source: BMC Microbiol. 2020 Jun 19;20:170. doi: 10.1186/s12866-020-01771-1 (PMC7304212; doi:10.1186/s12866-020-01771-1)
Supplement: Supplementary file 4 — Additional file 4 CRISPR/Cas9 generated alleles. Genes for which CRISPR/Cas9 alleles were generated are shown. Exons are indicated by green boxes and introns are indicated by black lines. Gene sizes are not to scale, but exon/intron size within genes is to scale. Relative location of the gRNAs is indicated by circles above the gene, and location of mutation is indicated in red (lines for deletions, triangles for insertions). All isoforms of K08D8.4 and W02A2.8 are shown. K08D8.4 mutations are predicted to result in loss of function of all isoforms; W02A2.8c may be expressed but is not differentially expressed between treatments. Mutation sequence and flanking sequence is shown on the right, with mutation sequence shown in red font (number in parentheses represents size of deletion). [file 12866_2020_1771_MOESM4_ESM.pdf]

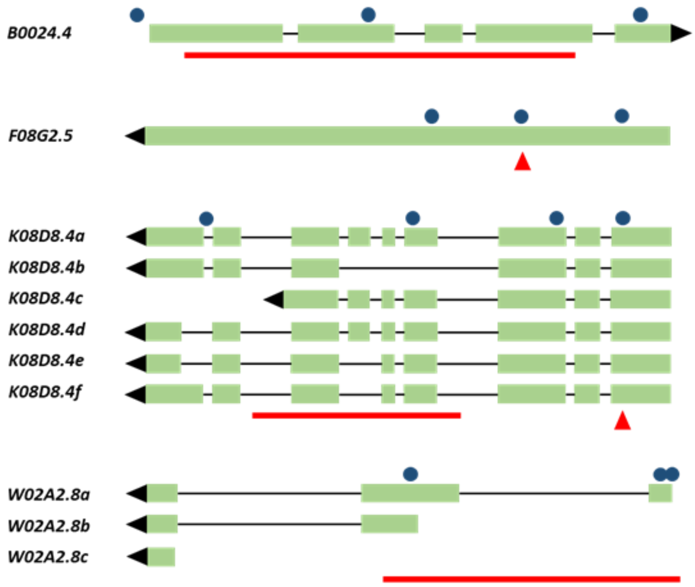

V:10297320..10298639
caaattctcaacttgaaagattttgatgtaaacactggcactaagatttc(1056bp)tagctg
gagaagtttttgtacagtattatatgattcgtaagtctcatttttgct

II:13833410..13833559
tcgagctcgtggagctggagattcagagatcggaagttccgaagaacgtggatattgacaccc
a(3bp)gtgtctctacgatcttgagaagttaacgtgtctacgatcttgagaagttaacgtggaaa
ccgtggagtcggaatatctatcttcatcgtccttatctgaaagttcaga

IV:12907960..12909763
caatgggggatttgtggcagtatatctgaaagtttatgtattaatgttata(997bp)cgtatga
tggcaaataaaagctattgtaagctattgtttataaaagctaaagc...cttgttcaccgttcggg
gtaggtacacttaattccagactcggagtcagttttcttgtaaaaaat

IV:13357200..13358639
tccacggatcgggacaagatcttgatgtacgtgattttggaattcgaagatga(1342bp)taa
caataaaatttgaatttgaacgaataagtgctctaaattttt
