# Supplementary figures and images for: Identification and characterization of differentially expressed genes in Caenorhabditis elegans in response to pathogenic and nonpathogenic Stenotrophomonas maltophilia
Source: BMC Microbiol. 2020 Jun 19;20:170. doi: 10.1186/s12866-020-01771-1 (PMC7304212; doi:10.1186/s12866-020-01771-1)

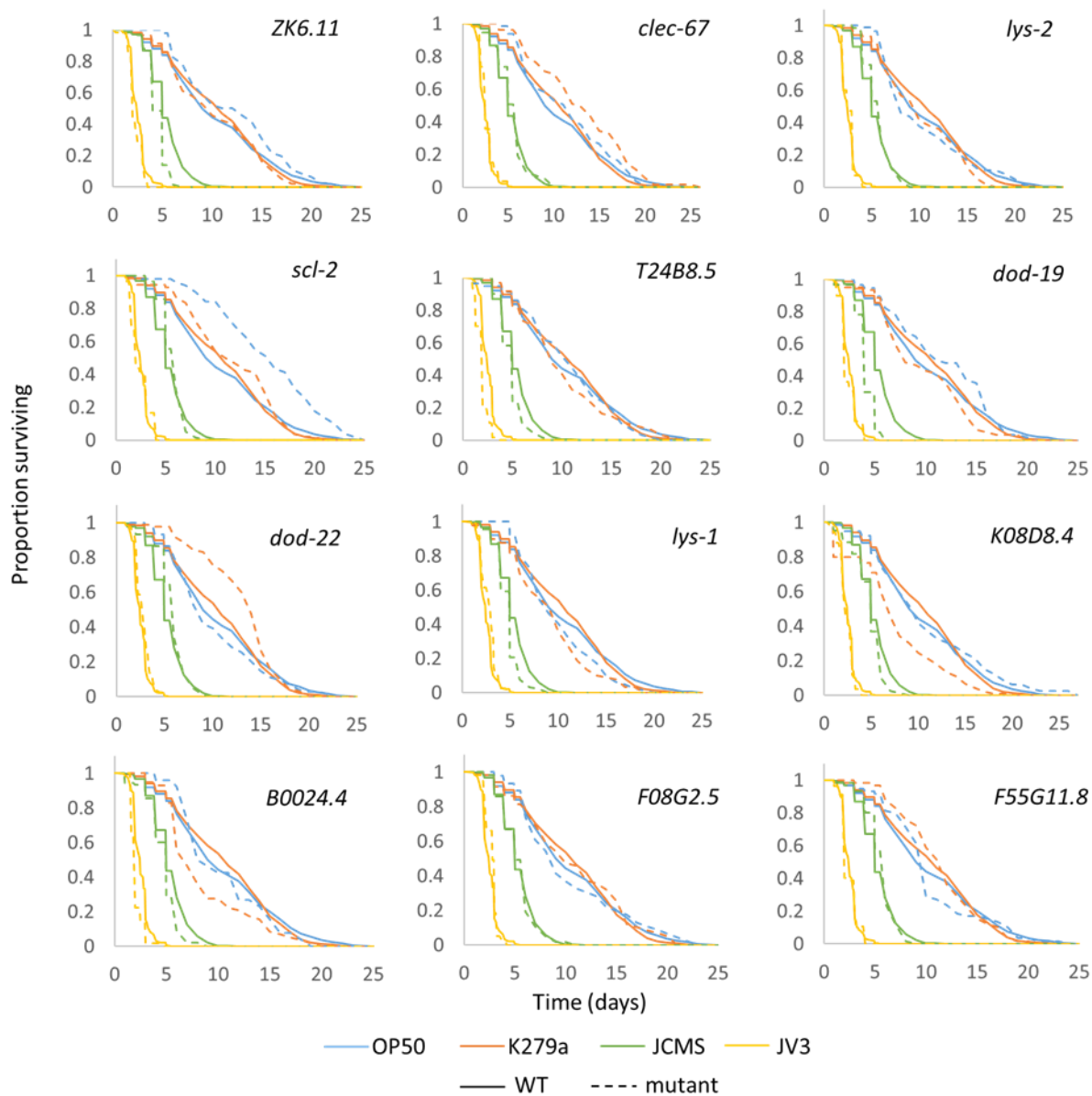

Supplement: Supplementary file 5 — Additional file 5 Mutations in CPSR genes result in a variety of survival patterns upon S. maltophilia exposure. Survivorship of wild-type nematodes and CPSR mutants on S. maltophilia JCMS, K279a, JV3, and E. coli OP50. Survival estimates were determined using Kaplan-Meier estimates generated in R. For these experiments, 10–12 worms were synchronized, picked onto each treatment bacterial lawn (3 plates per treatment/nematode combination) and the number of living worms was recorded daily. 2–3 replicates were completed for all bacterial and C. elegans strain combinations. Sample sizes, hazard ratios and p-values generated form Cox proportional hazards tests are shown in Table 3. [file 12866_2020_1771_MOESM5_ESM.pdf]

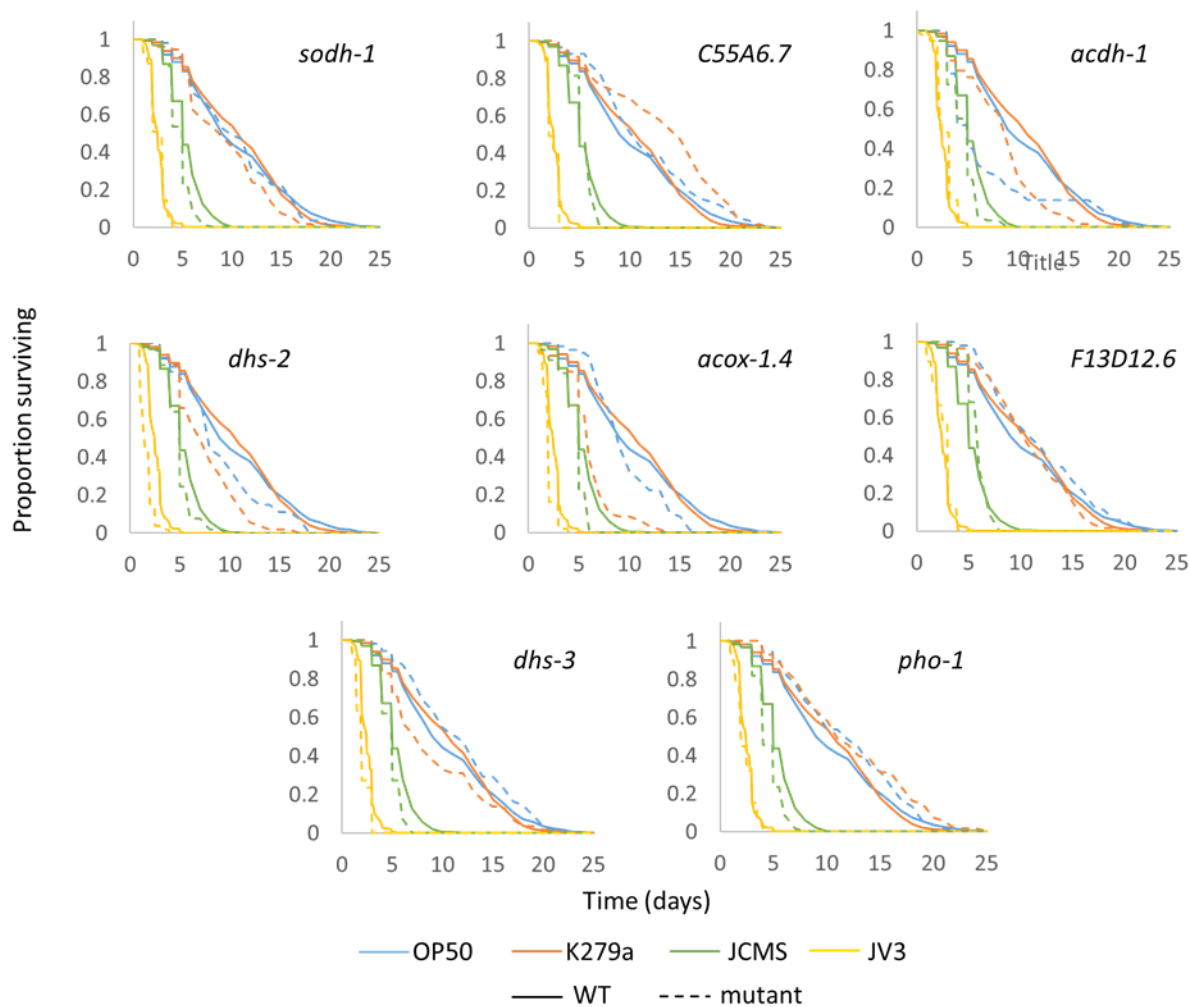

Supplement: Supplementary file 6 — Additional file 6 Mutations in VSR genes result in a variety of survival patterns upon S. maltophilia exposure. Survivorship of wild-type nematodes and VSR mutants on S. maltophilia JCMS, K279a, JV3, and E. coli OP50. Survival estimates were determined using Kaplan-Meier estimates generated in R. For these experiments, 10–12 worms were synchronized, picked onto each treatment bacterial lawn (3 plates per treatment/nematode combination) and the number of living worms was recorded daily. 2–3 replicates were completed for all bacterial and C. elegans strain combinations. Sample sizes, hazard ratios and p-values generated form Cox proportional hazards tests are shown in Table 3. [file 12866_2020_1771_MOESM6_ESM.pdf]

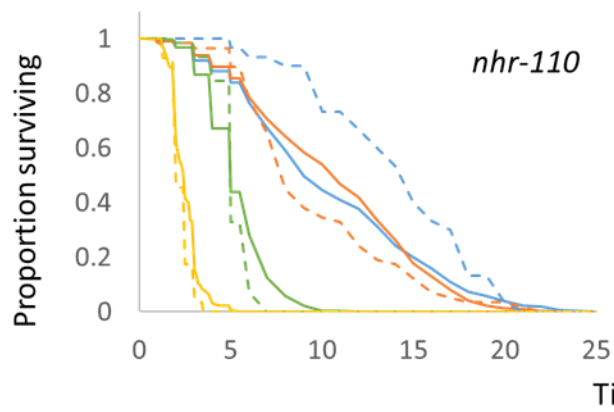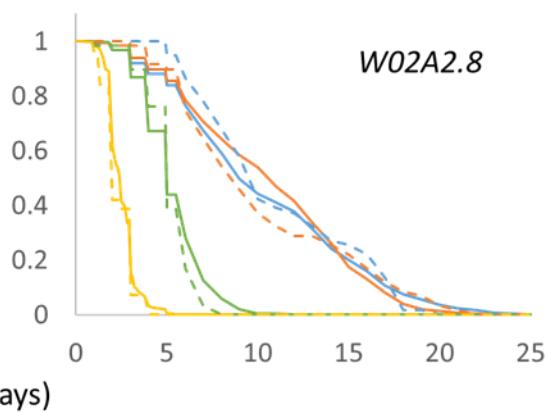

— OP50 — K279a — JCMS — JV3  
— WT ---- mutant

Supplement: Supplementary file 7 — Additional file 7 Mutations in JSR genes result in a variety of survival patterns upon S. maltophilia exposure. Survivorship of wild-type nematodes and JSR mutants on S. maltophilia JCMS, K279a, JV3, and E. coli OP50. Survival estimates were determined using Kaplan-Meier estimates generated in R. For these experiments, 10–12 worms were synchronized, picked onto each treatment bacterial lawn (3 plates per treatment/nematode combination) and the number of living worms was recorded daily. 2–3 replicates were completed for all bacterial and C. elegans strain combinations. Sample sizes, hazard ratios and p-values generated form Cox proportional hazards tests are shown in Table 3. [file 12866_2020_1771_MOESM7_ESM.pdf]

## CPSR

*K08D8.4*

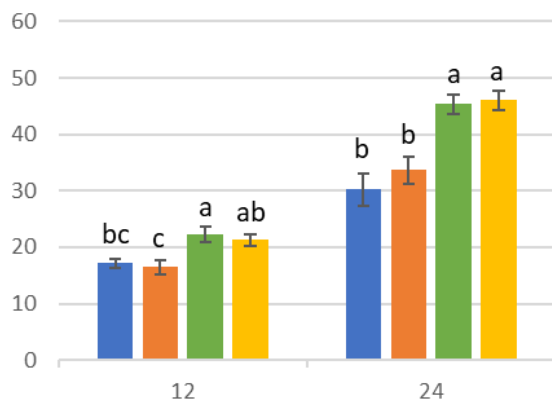

## VSR

*dhs-3*

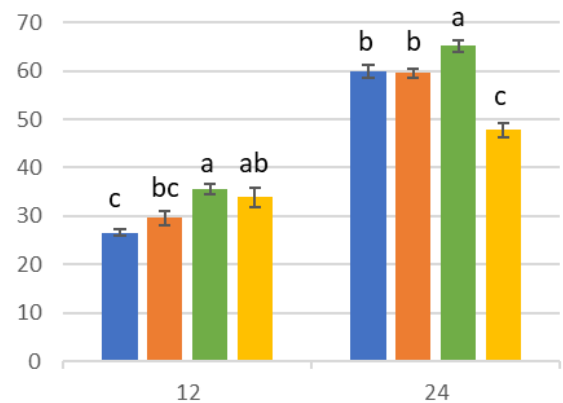

*F19B2.5*

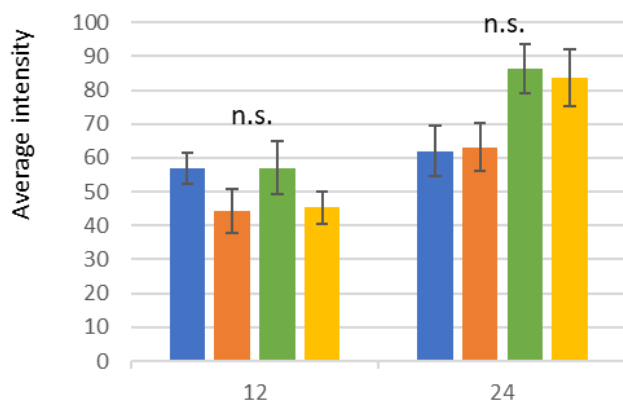

*acdh-1*

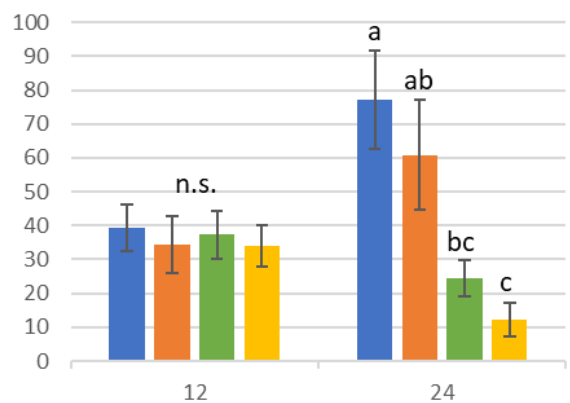

*T24B8.5*

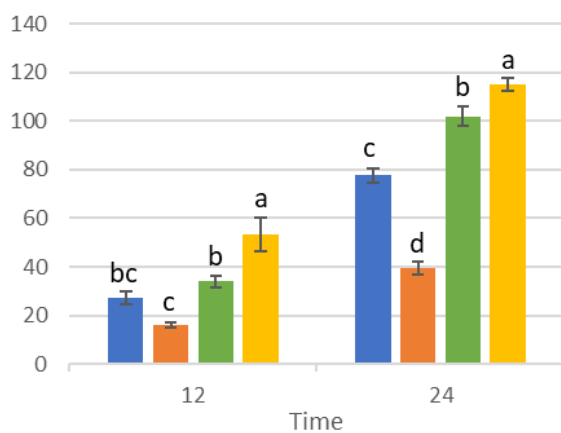

*sodh-1*

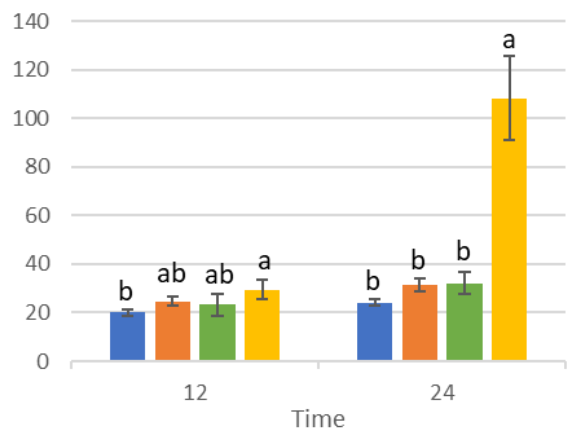

OP50

K279a

JCMS

JV3

Supplement: Supplementary file 8 — Additional file 8 Expression construct quantification at 12 and 24 h. Expression levels of several CPSR (T24B8.5, F19B2.5, K08D8.4) and VSR (sodh-1, acdh-1, dhs-3) genes using transcriptional or translational fluorescent protein fusions upon exposure to S. maltophilia or E. coli OP50. L4 worms containing expression constructs were moved to S. maltophilia K279a, JCMS, JV3, or E. coli OP50. After 12 and 24 h, images were taken of 10–18 worms for each treatment and expression construct, and average intensity was measured for each worm. Plots show mean average intensity and standard error for each time, treatment, and expression construct. Letters indicate significant differences across treatments for each time point (Tukey’s HSD). [file 12866_2020_1771_MOESM8_ESM.pdf]

# CPSR

*F19B2.5*

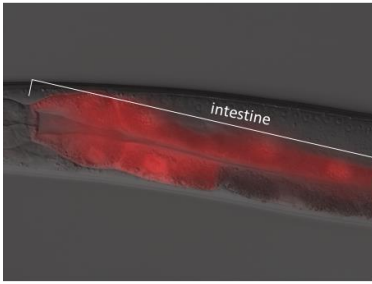

*T24B8.5*

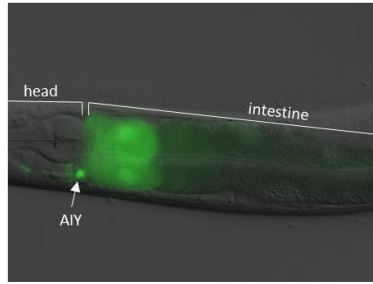

*K08D8.4*

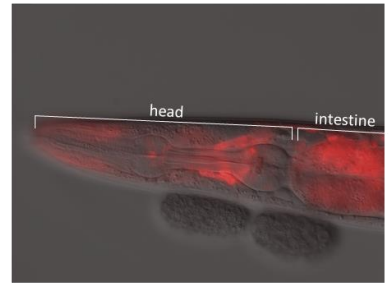

# VSR

*acdh-1*

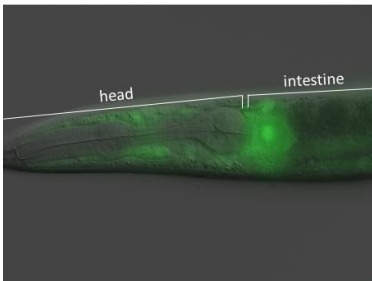

*dhs-3*

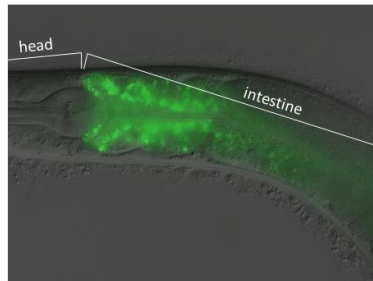

*sodh-1*

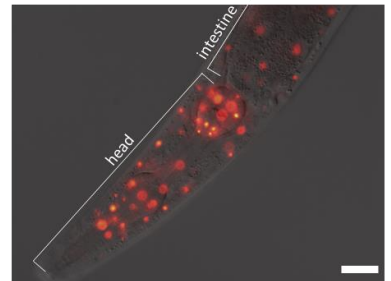

Supplement: Supplementary file 9 — Additional file 9 Expression construct localization. Expression of several CPSR (T24B8.5, F19B2.5, K08D8.4) and VSR (sodh-1, acdh-1, dhs-3) genes using transcriptional or translational fluorescent protein fusions upon exposure to E. coli OP50 at 400x in young adult worms. T24B8.5, F19B2.5 and dhs-3 are only expressed in the intestine, so the anterior intestine is shown. K08D8.4, acdh-1 and sodh-1 are also expressed in the head, which is shown. Brackets indicate head and intestine region on each worm. Unclosed brackets signify that region extends out of frame. Scale bar indicates 20 μm, anterior is to the left, and ventral is up for all pictures. Note that expression in the AIY interneuron (arrow) in the T24B8.5 transgenic strain is due to a ttx-3:GFP marker and not T24B8.5 expression and the sodh-1 expression construct contains a nuclear localization signal. [file 12866_2020_1771_MOESM9_ESM.pdf]
