# Supplementary material for: Identification and characterization of differentially expressed genes in Caenorhabditis elegans in response to pathogenic and nonpathogenic Stenotrophomonas maltophilia
Source: BMC Microbiol. 2020 Jun 19;20:170. doi: 10.1186/s12866-020-01771-1 (PMC7304212; doi:10.1186/s12866-020-01771-1)
Supplement: Supplementary file 10 — Additional file 10 CRISPR/Cas9 target gene primers. Forward and reverse primers for each gRNA were annealed and ligated into pRB1017. gRNA sequence (20 bp prior to the PAM site) are underlined for each primer, non-underlined bases are included in the primer sequence for proper ligation into BsaI digested pRB1017. Primers flanking the gRNA target loci were used to amplify DNA from candidate mutant worms to detect insertions or deletions based on amplicon size. Odd numbers of flanking primers listed for any gene were tested in combinations (forward primer was tested with each reverse primers). [file 12866_2020_1771_MOESM10_ESM.docx]

CRISPR/Cas9 target gene primers

| **Gene** | **gRNA Primers** | **Flanking Primers** |
| --- | --- | --- |
| *B0024.4* | 1 F: 5’-TCTTGCTAGAAATAGAAAGTTCGTT-3’ 1 R: 5’-AAACAACGAACTTTCTATTTCTAGC-3’ 2 F: 5’-TCTTGTCCTTCATACAACTTTACAG-3’ 2 R: 5’-AAACCTGTAAAGTTGTATGAAGGAC-3’ 3 F: 5’-TCTTGGGACGTCAAACTACATCACG-3’ 3 R: 5’-AAACCGTGATGTAGTTTGACGTCCC-3’ | F: 5’-CCATTTACACTCCTCCTC-3’ R: 5’-TTTACATCAAAATCTTTCAAGTTGAG-3’ R: 5’-AATTGTAATGATAAATGACGTGAATAG-3’ |
| *F08G2.5* | 1 F: 5’-TCTTGTCGAAGAATCCGTCTCCAAG-3’ 1 R: 5’-AAACCTTGGAGACGGATTCTTCGAC-3’ 2 F: 5’-TCTTGAAGATCGTAGAGACACCCAA-3’ 2 R: 5’-AAACTTGGGTGTCTCTACGATCTTC-3’ 3 F: 5’-TCTTGACAGAGATCGAAGAGAAAGT-3’ 3 R: 5’-AAACACTTTCTCTTCGATCTCTGTC-3’ | F: 5’-TAAAACCAGCACCTCTCACC-3’ R: 5’-ATCATCAGAGTCATCAGAAGAG-3’ |
| *K08D8.4* | 1 F: 5’-TCTTGATTAAGTGTACCTACCCGAA-3’ 1 R: 5’-AAACTTCGGGTAGGTACACTTAATC-3’ 2 F: 5’-TCTTGATATACATCGACCTTCCGT-3’ 2 R: 5’-AAACGACGGAAGGTCGATGTATATC-3’ 3 F: 5’-TCTTGGATTACTTGACTCTTCCGAA-3’ 3 R: 5’-AAACTTCGGAAGAGTCAAGTAATCC-3’ 4 F: 5’-TCTTGTTAAAAATAGAACAATACTT-3’ 4 R: 5’-AAACAAGTATTGTTCTATTTTTAAC-3’ | F: 5‘-CAGATAAATGTTCCTGAAGGC-3’ R: 5’-GCATCACTTGATTCACAGC-3’ R: 5’-CAGTGTTGGGAATGTTGTTG-3’ |
| *W02A2.8* | 1 F: 5’-TCTTGAGCGGATTCCCGATTCACGA-3’ 1 R: 5’-AAACTCGTGAATCGGGAATCCGCTC-3’ 2 F: 5’-TCTTGGGAAGCGTCCTCATTCAACG-3’ 2 R: 5’-AAACCGTTGAATGAGGACGCTTCCC-3’ 3 F: 5’-TCTTGATTTTGACCCCCCATGACGG-3’ 3 R: 5’-AAACCCGTCATGGGGGGTCAAAATC-3’ | F: 5’-GTATTTCTTGTGATTCTAGAGTCACC-3’ R: 5’-GGAAGAAAATAGCGGAATAGGTTAC-3’ R: 5’-AACAACCAAAGACGAACCTC-3’ |
